# A Bacteriophage-Related Chimeric Marine Virus Infecting Abalone

**DOI:** 10.1371/journal.pone.0013850

**Published:** 2010-11-05

**Authors:** Jun Zhuang, Guiqin Cai, Qiying Lin, Zujian Wu, Lianhui Xie

**Affiliations:** 1 Key Laboratory of Plant Virology of Fujian Province, Institute of Plant Virology, Fujian Agriculture and Forestry University, Fuzhou, China; 2 Key Laboratory of Biopesticide and Chemical Biology, Fujian Agriculture and Forestry University, Ministry of Education, Fuzhou, China; The J. Craig Venter Institute, United States of America

## Abstract

Marine viruses shape microbial communities with the most genetic diversity in the sea by multiple genetic exchanges and infect multiple marine organisms. Here we provide proof from experimental infection that abalone shriveling syndrome-associated virus (AbSV) can cause abalone shriveling syndrome. This malady produces histological necrosis and abnormally modified macromolecules (hemocyanin and ferritin). The AbSV genome is a 34.952-kilobase circular double-stranded DNA, containing putative genes with similarity to bacteriophages, eukaryotic viruses, bacteria and endosymbionts. Of the 28 predicted open reading frames (ORFs), eight ORF-encoded proteins have identifiable functional homologues. The 4 ORF products correspond to a predicted terminase large subunit and an endonuclease in bacteriophage, and both an integrase and an exonuclease from bacteria. The other four proteins are homologous to an endosymbiont-derived helicase, primase, single-stranded binding (SSB) protein, and thymidylate kinase, individually. Additionally, AbSV exhibits a common gene arrangement similar to the majority of bacteriophages. Unique to AbSV, the viral genome also contains genes associated with bacterial outer membrane proteins and may lack the structural protein-encoding ORFs. Genomic characterization of AbSV indicates that it may represent a transitional form of microbial evolution from viruses to bacteria.

## Introduction

Marine viruses are unarguably abundant and diverse members of microbial communities in the marine biosphere [1], [2]. They have major roles in the oceanic carbon and energy cycles and cause diseases in a wide range of organisms [3]–[5]. In late 2005, an outbreak of abalone fatal disease, referred to as abalone shriveling syndrome (AbSS), spread to several fisheries in Fuzhou, China. Abalones in all life-stages can suffer from the disease. The infection was characterized by pedal muscle atrophy and lesion of mantle tissue with nigrescence (Figure S1). Accompanied by a reduction in feeding, most infected abalones fall from the reef and die. These signs were similar to withering syndrome in the black abalone, previously reported to be associated with *Rickettsiales* bacteria [6]. Herein, we prove from experimental infection of healthy abalone that an unclassified, novel virus can cause AbSS, named as AbSS-associated Virus (AbSV). In infected abalone, the cytopathic changes (e.g. necrosis etc.) were observed and accompanied with the modifications of host macromolecules (i.e. hemocyanins and ferritins). The complete viral genomic sequence reveals that putative genes in the virus are linked to bacteriophages, eukaryotic viruses, bacteria and endosymbionts. In addition, the virus's mosaic genome presents a gene organization similar to the majority of tailed phages.

## Materials and Methods

### Sample collection and experimental virus infection

Diseased abalones (*Haliotis*. *diversicolor aqualitis*) associated with AbSS were collected from culture fisheries in Fuzhou, China in November, 2005. To determine the infectivity of AbSV, we carried out artificial infection trials as follows. Before infection, healthy abalones (collected from the Test Base for Fishery Technical Extension Station of Fujian Province) were raised in the lab for 7 days, and then divided into two equal groups. One group of healthy abalones (experimental) were inoculated with a 0.22 µm-pore-diameter filtered AbSV sample from naturally infected abalone under a 0.02MPa vacuum, while the control group was treated with sterile sea water (filtered) in parallel. Control and experimental groups were stocked in separate aquaria for the duration of experiment. Their clinical signs (e.g. feeding, adhesion power, activity, etc.) were monitored and recorded daily. Individuals exhibiting symptoms were collected, and some were preserved in fixative (1.5% glutaric dialdehyde mixed with 1.5% paraformaldehyde) for ultrathin electron microscopy sectioning. For further virus infection confirmation, other samples were subjected to southern/dot blot hybridization for virus detection and virus purification.

To illuminate AbSV pathogenicity, another experimental infection was performed through direct injection of viral DNA into the abalone pedal muscle. Briefly, adult individuals of abalone (*H. discus hannai*) were kept in a 100 L artificial seawater aquarium at 20°C. After 1 week, the individuals were divided into two equal groups. One group of individuals was inoculated with purified AbSV genomic DNAs through syringe injection, while the negative control group was inoculated with artificial sea water in parallel. The artificial experiments were replicated twice; filtered sea water was used in the first experiment, and artificial sea water was used in the second one.

### Purification of AbSV

Viral purification was carried out according to a previously described technique with the following modifications [7]. A 200 g batch of infected abalone was ground in liquid nitrogen, then mixed with 1 L of TN buffer (0.1 M Tris-Cl, 0.15 M NaCl, 5 ng/mL PMSF, pH 8.0) and homogenized using an electric homogenizer. The suspension was sieved successively through four-layered gauze to remove large debris, and then centrifuged at 5500 *g* for 10 min. Triton X-100 was added to the supernatant to 1% final concentration, homogenized in a magnetic stirrer for 2 h at 4°C, then centrifuged at 12000 *g* for 30 min. Discarding the precipitate, PEG-6000 and NaCl were added to the upper supernatant to 7% and 3% final concentrations, respectively, stirred again 4°C for 2 hr, and then centrifuged at 12000 *g* for 30 min. The pellet was resuspended with PBS (pH 7.2) and centrifuged 12000 *g* for 30 min. The supernatant containing the virus was ultra-centrifuged in a Beckman type Ti45 rotor for 1.5 hr at 40000 rpm at 4°C. The virus pellet was first gently washed to remove the remaining salt with PBS (pH 7.2), then resuspended in 4 ml PBS, and finally centrifuged at 12000 *g* for 10 min. Aliquots of the supernatant were stored at −20°C. Morphology of the isolated virus particles was determined by electron microscopy using a negative-staining methodology with 2% phosphotungstic acid as described below.

In addition to PEG-mediated ultracentrifugation, two other methods were carried out for AbSV purification, sucrose cushion ultracentrifugation and sucrose gradient ultracentrifugation. The three methods shared the pretreatment before ultracentrifugation. For sucrose cushion ultracentrifugation, after Triton X-100 was added to 1% final concentration, the suspension was homogenized in a magnetic stirrer for 2 hr at 4°C, and centrifuged at 12,000 *g* for 30 min. The supernatant was collected by aspiration and then layered on a 20% sucrose cushion and centrifuged at 40,000 rpm (type Beckman Ti45) for 1.5 hr at 4°C. The virus pellet was resuspended in 2 ml PBS (corresponding to 100 g infected abalone). For sucrose gradient ultracentrifugation, after PEG-mediated centrifugation, this virus suspension was added to the top of a sucrose gradient ranging from 10%–50%. The gradients were spun in a Sw40 rotor for 8 hr at 30,000 rpm at 4°C. The virus particles band was collected and diluted with PBS buffer and pelleted by centrifugation at 40,000 rpm (type Beckman Ti45) for 1.5 hr at 4°C. The virus pellet was resuspended in 2 ml PBS (corresponding to 100 g infected abalone).

### Electron microscopy and histopathologic examination

Viral pellets were resuspended in autoclaved buffer (100 mM Tris-HCl, 100 mM NaCl, 50 mM MgCl_2_, pH 7.5). Approximately 10 µL virus suspension was dripped onto a 200 mesh copper grid coated by a formvar membrane, negatively stained using filtered (0.22 µm) 2% phosphotungstic acid (PTA), and dried at room temperature.

For ultrathin sectioning, several small batches of infected-virus tissues were incubated at 4°C for 16 hr in a primary fixative containing 3% (V/V) glutaric dialdehyde and 1.5% paraformaldehyde in 0.1 M sodium phosphate. After three washes in 0.1 M phosphate-buffered saline (PBS, pH 7.2) at room temperature, the tissue was transferred to a secondary fixative containing 1%(W/V) osmium tetroxide and 1.5% (W/V) potassium ferrocyanide in 0.1 M PBS (pH 7.2) at 4°C for 1.5 hr. The tissue was then washed twice in 0.1 M PBS (pH 7.2) and immersed in 50% ethanol for 10 min before transfer to saturated uranyl acetate in 70% ethanol for 16 hr at 4°C. The tissue was dehydrated in an acetone series and embedded in resin for ultrathin sectioning. Ultrathin sections were counterstained with uranyl acetate and lead citrate before examination. Observations were performed using a JEM-1010 transmission electron microscope.

### Preparation of viral DNA

To remove contaminated DNA, the virus suspension was first digested at 37°C for 20 min with a limited amount of DNaseI (Promega). The enzyme was inactivated by heating for 10 min at 75°C. Then the virus particles were incubated with 1% sodium dodecyl sulfate at 65°C for 20 min to release genomic nucleic acid. An equal volume of 3 M potassium acetate (pH 5.2) was then added, mixed, and placed at −20°C for 10 min. After alcohol precipitation, the virus DNA pellet was resuspended in TE (pH 8.0). Finally, the viral DNA was further purified using an UltraClean DNA Purification Kit (MBI). A conventional PCR was performed with specific primer for bacterial 16S rDNA (27F: 5′-AGAGTTTATCCTGGCTCAG-3′, 1510R: 5′-GGTTACCTTGTTACGACTT-3′) to confirm whether the viral DNA was slightly contaminated by bacterial cells and/or DNA.

### Sequencing of AbSV genomic DNA

Two partial restriction libraries were generated by digestion of AbSV genomic DNA with *Bam*HI (TaKaRa) and *Hae*III (TaKaRa), followed by ligation into pUC19. Random DNA fragments larger than 1.0 kb were cloned and used in dideoxy sequencing reactions. Reaction products were analyzed on an ABI PRISM 3730xl automated DNA sequencer (Applied Biosystems, Foster City, Calif.). Gaps in the AbSV sequence were closed by PCR amplification using viral genomic DNA as the template. Each 25 µl reaction mix contained 5 ng of AbSV genomic DNA, 10 pmol of each primer, 0.2 mM concentrations of each of the four deoxynucleoside triphosphates, 2.0 mM magnesium chloride, 1 U of LA-*Taq*, and 1×LA buffer A (TaKaRa). Amplifications were carried out in a T3 Thermoblock thermal cycler (Biometra) using an initial denaturation step of 94°C for 1 min, followed by 30 cycles of 97°C for 15 s and 66.5°C for 7 min. Five pairs of primers were used for genome closure as follows: 50F2: 5′-GACGATGCTGCTGCTGAGGAGATTGCTAAGACAC-3′, 59R2: 5′-GATGTGGTGGTGGTTGC CCACTTAGATCGATTAG-3′; 50R1:5′-CCCCGGTCGATCATCTCTTGAGTCATTGCCT-3′, 2.5KR2: 5′-GGGCGTGACTTTAGCCACTGGCCTAGAAG-3′; 2.3KP2: 5′-CGATCGAGGTATTTC TCATTGCTCTCTTCCGAT-3′, 59F1: 5′-AGAGGACAGGACTCTTGACCACCTAGTAGCCG-3′; 2.3KP1: 5′-CTCGGACAACAGCATTGTGTTAACTCATGAGCC-3′, S58R: 5′-TGCAACTTGGGC AGCGTTAGTGGCTGTTTG-3′; 2.5KF2: 5′-CTAGTGGTGATTATTACTATGCAGTGAGCGGC-3′, S58L: 5′-ATGGCCTCACAGGTGATCCAATTATTCAAATTC-3′. The PCR products were retreated with Exonuclease I and then precipitated with 75% (v/v) ethanol. The resulting DNA pellet was suspended in an appropriate volume of TE, and the purified PCR products were sequenced as described above.

The validity of the genome assembly was assessed by comparing the agarose gels' restriction patterns to the computer-predicted restriction patterns of the consensus sequence. Single and double digestion patterns were used. This comparison confirmed the predicted restriction fragment lengths of AbSV genomic DNA, as well as its circular topology.

### Sequence analysis

Presumptive ORFs were defined with ORFfinder and GeneMark.hmm 2.0 [8]. Homology of the ORF-encoded protein sequences were assessed through BLASTP and tBLASTn searches against the NCBI non-redundant protein database (http://www.ncbi.nlm.nih.gov). If the best matching functional homologues for AbSV ORF exhibited an E-value smaller than 5e^−4^, we have assigned the homologues to the AbSV ORFs. Functional motifs and conserved domains were identified by searches against the Prosite database (http://cn.expasy.org/tools/scanprosite/), the Pfam protein family database (http://pfam.sanger.ac.uk/search), and the Superfamily database (http://supfam.mrc-lmb.cam.ac.uk/SUPERFAMILY). Homologues of AbSV proteins in the environmental sequence data were detected by searching the NCBI environmental data set using BLASTP. The genome map was generated with CGView [9]. Clustalx1.83 was used to construct multiple alignments. Phylogenetic analyses were conducted with MrBayes 3.2 [10], MEGA 4 [11], or Phylip 3.67 [12].

### Preparation of labeled probe and Southern/dot blot

On the basis of the sequence of a PCR fragment, a pair of specific primers (S58-11-L: 5′-GCCTCACAGGTGATCCAA-3′, S58-11-R: 5′-TGCAACTTGGGCAGC GT-3′) was designed for a 560 bp product as a DIG-labeled probe. The probe containing DIG-labeled dUTP (Roche) was synthesized according to the manufacturer's protocol. Genomic DNA from healthy and diseased abalones were extracted by lysis with proteinase K and SDS as described previously [13]. The lysate of virus-like particles treated by SDS and the other samples were all loaded in a 0.8% agarose gel, and then the DNA was transferred onto nylon Hybond-N^+^ membranes (Amersham), as described previously [14]. The recombinant plasmid corresponding to the cloned PCR fragment and DNA extracted from healthy abalone served as positive control and negative controls, respectively.

### RNA isolation and reverse transcription

Total abalone RNA was purified with Mollusc RNA Kit (Omega) and treated with RNase-free DNaseI (Takara) to remove genomic DNA contamination. One microgram of total RNA was used for reverse transcription reaction with H Minus M-MuLV Reverse Transcriptase (Fermentas) and 3′-terminal primer, according to the manufacturer's protocol. PCRs were performed with Ex-Taq (Takara). Primer sequences are shown in Table S1.

### 2-DE analysis of hemocyanin-like protein

A 30 µL vial of hemocyanin-like resuspension was thawed at room temperature, and then SDS lysis buffer (1% final concentration) was added and mixed. The solution was incubated in a boiling water bath for 5 min and centrifuged at 12000 *g* for 5 min at 4°C. Soluble proteins in the supernatant were first precipitated in freezing (−20°C) acetone, and then resuspended in a rehydratation solution buffer [5 M urea, 2 M thiourea, 4% (w/v) CHAPS, 60 mM DTT, 0.2% (v/v) Bio-Lyte (Bio-rad)]. IPG DryStrips (7 cm, pH 4–7, Bio-rad) were rehydrated for 12 hr in 150 µl rehydratation buffers containing 50 µg of proteins and isoelectric focusing (IEF) was carried out at 4000 V according to the manufacturer's protocol. Prior to the second-dimension electrophoresis, strips were equilibrated twice in 2 ml equilibration buffer [20% (v/v) glycerol, 2% (w/v) SDS, 6 M urea, 0.375 M Tris/HCl, pH 8.8] for 10 min. This equilibration buffer was supplemented with 130 mM DTT for the first equilibration and with 135 mM iodoacetamide for the second one. The strips were then embedded in a 0.5% agarose and the proteins resolved by 12.5% SDS-PAGE at 80 V for 10 min, followed by 120 V for 1.5 hours. Following electrophoresis, the gels were processed by traditional Coomassie brilliant blue R-250 staining. The signal of stained gels was captured with the Gene Genius Bio Imaging System (Syngene, Frederick, MD, USA).

### In-gel digestion and MS identification

Protein spots were excised from the gels and placed into a 96-well microtitre plate. Gel pieces were de-stained with a solution of 50 mM ammonium bicarbonate in 50% acetonitrile for 30 min at 37°C. The samples were washed twice with deionized water and shrunk by dehydration in acetonitrile. The samples were then swollen in digestion buffer containing 25 mM ammonium bicarbonate and 12.5 ng/µl trypsin at 4°C. After 30 min incubation, the gel samples were digested for at least 12 hrs at 37°C. Peptides were then extracted twice using 0.1% trifluoroacetic acid in 50% acetonitrile. The dried samples were redissolved in 0.8 uL 50% acetonitrile, 0.1% trifluoroacetic acid containing 5 mg/mL α-cyano-4-hydroxycinnamic acid (Sigma, USA). Then the solution was spotted on a stainless steel target with 192 wells (Applied Biosystems, USA) and allowed to air dry. Spectra were collected by using an ABI4700 matrix-assisted laser desorption ionization tandem time-of-flight (MALDI-TOF/TOF) mass spectrophotometer (Applied Biosystems, USA). The resulting MS/MS ion data were performed *de novo* sequence analysis and searched through the MASCOT search engine (www.matrixsciences.com) for identification.

Similarly, viral protein spots stained with Coomassie brilliant blue R-250 were excised from the gel, reduced, and alkylated, and then an in-gel trypsin digestion was performed. The resulting peptides were extracted from the gel, dried, and dissolved in 0.1% trifluoroacetic acid and injected onto a desalting reverse-phage column via an auto-sampler. Peptide sequencing was performed on a quadrupole time-of -flight (Q-TOF) mass spectrometer (Micromass, Manchester, United Kingdom) equipped with a nanoflow electrospray ionization (ESI) Z-spray source. The precursor ions were selected with a quadrupole analyzer, and fragmentation took place in a hexapole collision gas cell with argon as the collision gas. The data acquisition and deconvolution was performed with MassLynx 4.0 software.

### Iron detection in virus preparation

Virus suspensions in native polyacrylamide gels were stained with Perl's staining, which was carried out by incubation in 2% K_4_Fe (CN)_6_ (Postassium ferrocyanide)/2% HCl (hydrochloric acid) after rinsing with ddH_2_O. The stained gel was analyzed using a Typhoon scanner.

## Results

### Optimization of AbSV isolation for DNA extraction

Extraction of viral DNA for sequencing requires a large amount of virus suspension containing a high concentration of virus. We, therefore, optimized the purification method for AbSV DNA. Despite its removal of some impurities, the sucrose gradient ultracentrifugation was unfit for further purification of AbSV because the partial viral DNA was prone to truncation after this purification method. The yield of viral DNA obtained from sucrose cushion ultracentrifugation was also significantly reduced compared to that obtained after PEG-mediated ultracentrifugation. Therefore, because of the preserved integrity and high yield of viral DNA obtained, we chose PEG-mediated ultracentrifugation for large-scale virus preparation.

### Pathogenicity of AbSV

**Figure 1:**
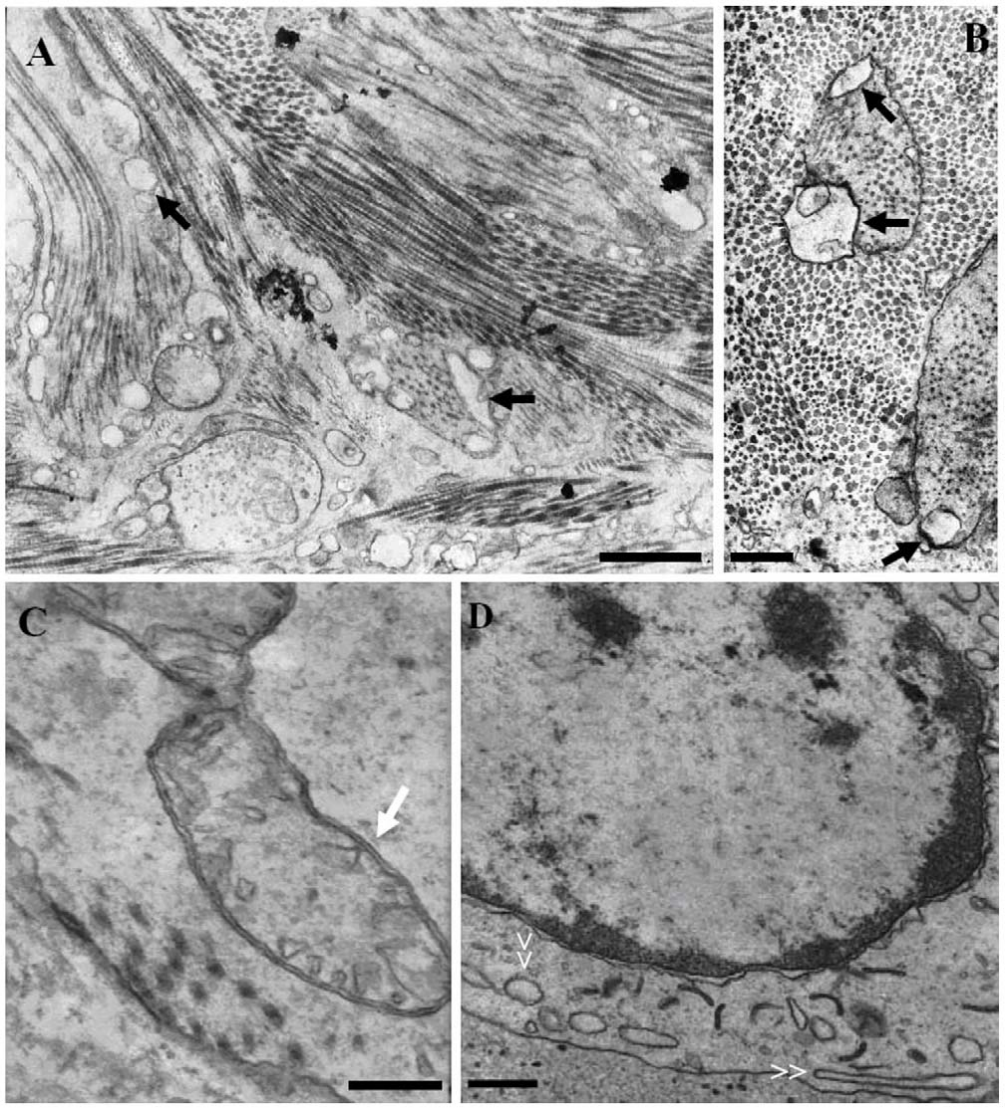
Pathological ultrastructures of AbSV-infected abalone tissues (pleopod and alimentary canal). The myocytes of pleopods displayed diffuse alveolar damage (black arrows) in vertical (**A**) and cross section (**B**) of myofilaments. In hepatopancreas cells, the mitochondria cristae appeared to be fragmented (**C**) and dissolved accompanying marginalization of chromatin and extranuclear aggregates which might originate from fractured pieces (white double arrows) of ERs or other organelles (**D**). Bars represent 1 µm in panel **A**, 200 nm in panel **B** and **C**, and 500 nm in panel **D**.

Artificially AbSV-infected abalones were collected and examined over the course of viral infection. Histologically, pleopods with gross lesions had diffused alveolar damage, marked by necrosis of myofilament (Figures 1A and 1B). Evidence of cellular necrosis induced by the virus was also present in infected alimentary canal cells. In particular, the mitochondria cristae appeared to be fragmented (Figure 1C), and the marginalization of chromatin was obviously visible along with the extranuclear aggregates which might have originated from fractured pieces of the endoplasmic reticulum (ER) or other organelles (Figure 1D). By just 1 day post-inoculation (d.p.i.), the inoculated abalone individuals exhibit reduced feeding behavior. After 3 d.p.i., the pedal muscle adhesion of most infected abalone to aquarium substrate was greatly reduced.

As illustrated by southern/dot blot hybridization (Figure S2), the genomic DNA of the AbSV which replicated in the artificially infected abalones and produced clinical signs and lesions indistinguishable from those in naturally infected abalones was the same as that found in naturally infected abalone populations. Additionally, RT-PCR analysis revealed that transcripts of viral genomic DNA only appeared in infected individuals and were undetected in healthy abalone (Figure S3). Thus, this virus fulfills Koch's postulates as the primary aetiological agent of AbSS. Nevertheless, this does not rule out the possibility that other pathogens including bacteria (e.g. *Pseusomonas fluorescens*) [15], may exacerbate the disease in some AbSS cases.

### Modified hemocyanin and ferritin identified by MS analysis in AbSV

**Figure 2:**
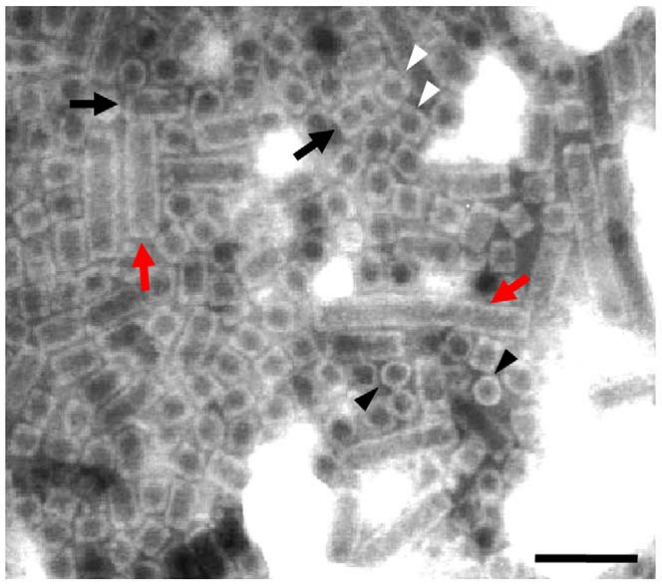
TEM of particles in an AbSV suspension. Novel particles are shown from the side view as long flexible/rigid rectangles (red arrows) and short rectangles (black arrows). The circles (black arrowheads) and cubes (white arrowheads) from the top view and side views, respectively, are all similar to typical hemocyanin architectures. Scale bar, 100 nm.

Novel particles, which appear as rectangles of 30×30∼150 nm (and even longer) from the side and as circles of ∼30 nm in diameter from top (Figure 2; Figure S4D), were isolated from the same diseased abalones (*Haliotis diversicolor aquatilis*) used for viral genomic DNA preparations. Morphologically, the circular particles are identical to abalone hemocyanin, and the long rectangular particles are indistinguishable from reassociated hemocyanin-like particles (appearing as helical tubules or longer multidecamers) *in vitro* as described by Harris *et al*. [16]. To further characterize the exact protein components of AbSV suspensions and examine their hemocyanin-like properties, we analyzed the constituent proteins (45 to 100 kD) from purified AbSV suspensions via mass spectrometry. Three important points emerged from this analysis.

**Figure 3:**
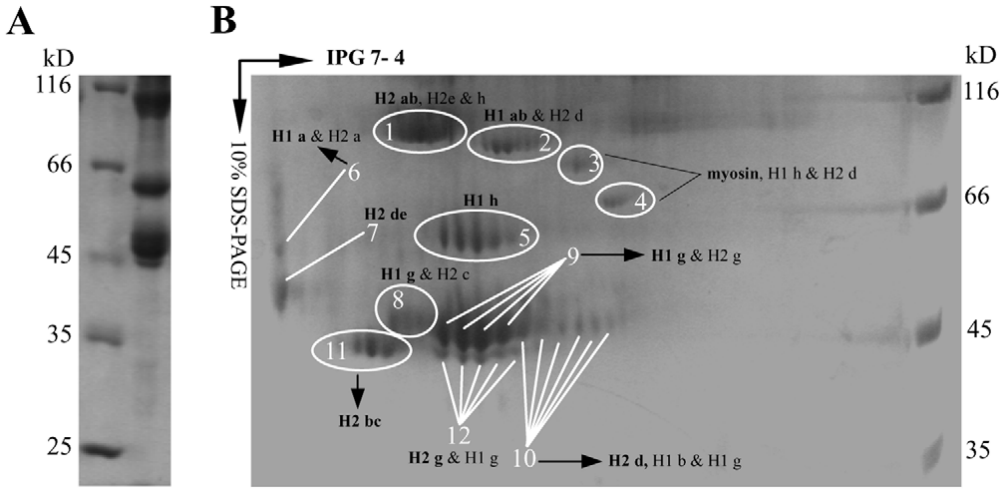
The electrophoresis profiles of hemocyanin-like proteins. The denatured proteins were separated in a 12.5% SDS-PAGE gel (A), or on isoelectric focusing (4–7) IPG strips and then separated in the second dimension on a 10% SDS-PAGE (B). The numbered spot proteins with equivalent MW were identified as the same protein isoforms by MALDI-TOF/TOF mass spectrometry. These structural proteins, which were all associated with abalone hemocyanin subunits [type 1 or type 2, i.e. H1or H2 consisting of a corresponding set of functional units (FUs) (termed H1 a to H1 h, or H2 a to H2 h)], were composed of the fragmented hemocyanin subunits or splicesome of different FUs between subunits. The corresponding FUs of peptide sequences in different spot proteins were referred, and the bold represented dominant component in the protein(s).

Firstly, most proteins in purified AbSV suspensions were definitely identified as fragmented or modified abalone (host) hemocyanin subunits type 1 and type 2 (H1 and H2, ∼400 kD respectively), which act as extracellular oxygen transport proteins in abalone cells. Hemocyanin subunits can exist as either as eight functional units (FUs) connected by linker peptides in a linear order [17]–[21], or as a splicesome of different FUs or FUs and other proteins (e.g. myosin) (Figure 3; Figure S5; Table S2). Six of the proteins spots (spots 2, 6, 8, 9, 10 and 12, Figure 3) observed in AbSV suspensions were composed of splicesomes of abalone hemocyanin subunits 1 and 2; their dominant composition present to H1 FU-ab, H1 FU-a, H1 FU-g, H1 FU-g, H2 FU-g and H2 FU-d, respectively. Additionally, one peptide (m/z 1215.7) exhibits a single amino acid deletion from abalone hemocyanin subunit 1(Table S2). By MASCOT search and homology analysis, the hemocyanin-like proteins in the 45 to 100 kD region (shown in Figure 3) contain a partial sequence of hemocyanin subunit 1 FU-a, -b, -d, -g and -h, and hemocyanin subunit 2 FU-a, -b, -c, -d, -e, -g and -h (Table S2). Several FUs of hemocyanin subunits and modified ones in AbSV suspension most likely play architectural roles in the formation of long, flexible or rigid hemocyanin-like multidecamers or polymers observed in infected abalone but not in healthy abalone (Figure S6), owing to ∼45% identity of protein sequence in different FUs [28] and reassociation ability of dissociated hemocyanin subunits [16].

Secondly, individual spot proteins (spots 1, 4 and 5, Figure 3) were identified as virus or phage structural protein-related sequences. The inferred sequence (WAI/LKPFNR) of a peptide (m/z 1031.58), which is detectable in all trypin-digested extracts of protein spots 1 and 5, shares homology with the structural sequences from phages and viruses, such as mimivirus (Table S2), but does not match with host abalone hemocyanin subunit sequences.

Thirdly, ferritin subunits were also unambiguously identified by electrospray ionization quadrupole time-of-flight (ESI-Q-TOF) MS and MALDI-TOF/TOF MS (Figure S7; Table S3) in AbSV viral suspensions, and a vast amount of iron was detected by Prussian blue staining (Figure S7C). Intriguingly, the unambiguously identified sequence (^N^IVLQDVGARP^C^) of peptide with m/z 1223.75 shares homology with the identical sequence of a ferritin subunit within diverse abalone species and other mollusks (Figure S8). This same sequence has similarity to ^136^IVLQDIGGKP^145^ from the tail fiber domain of Enterobacteria phage T4, suggesting that abnormal modification of ferritin subunit also appeared in the infected abalone.

### AbSV represents a novel, unclassified virus family

**Figure 4:**
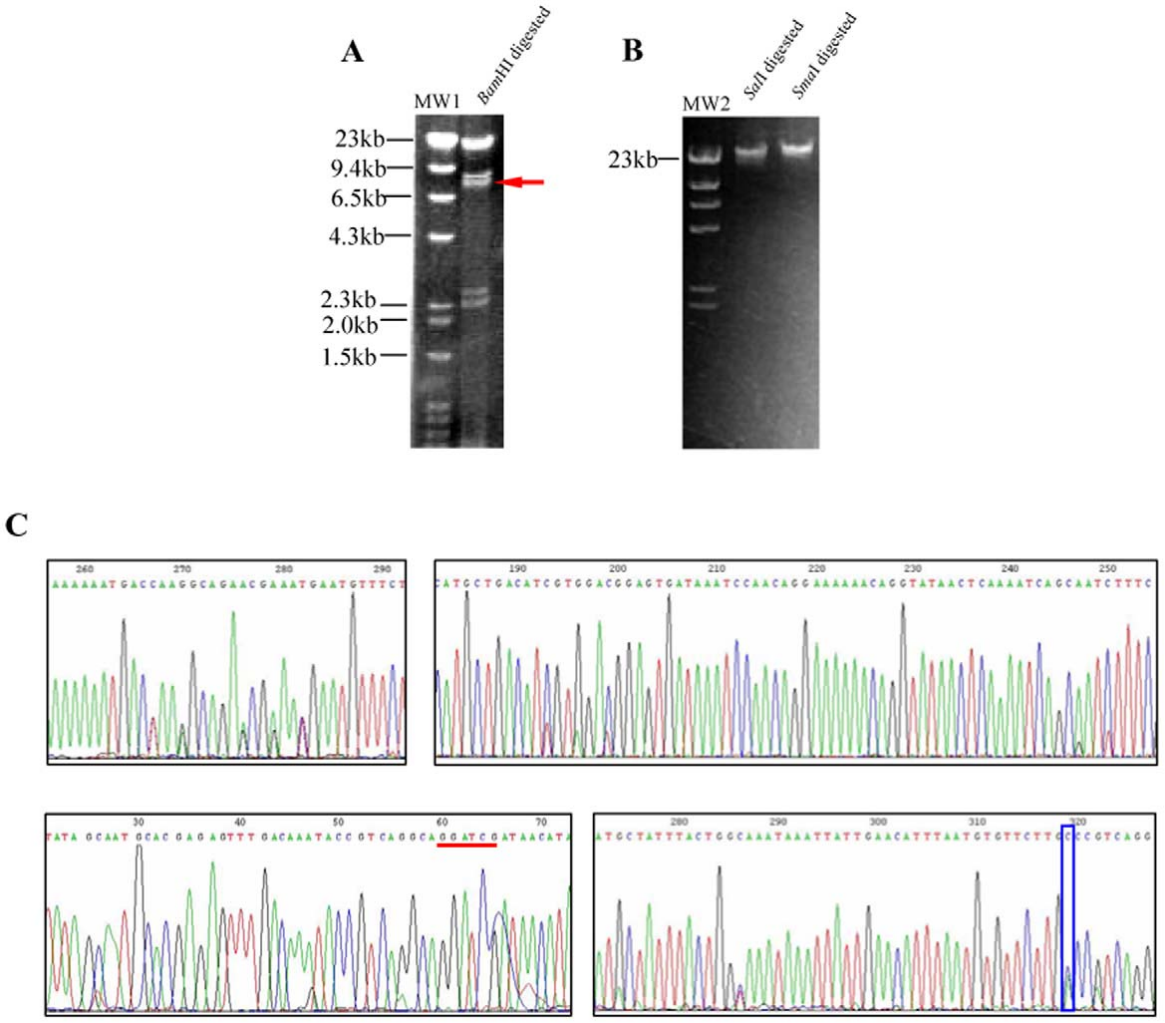
Restriction enzyme digestion of the AbSV genome and representative site mutations in genome. (A) Patterns of a circular genome digestion with 5 bands of 2.35 kbp, 2.6 kbp, 7.8 kbp, 8.5 kbp and 21 kbp, respectively, produced by *Bam*HI digestion. Site mutation resulted in one additional band (indicated by red arrow, ∼7.8 kbp). (B) Linear genome resulting from digestion with *Sal*I and *Sma*I, both of which have only cut site in the genome. MW1: Lambda *Hin*d III marker +100 bp ladder marker (TaKaRa), MW2: Lambda *Hin*d III marker (TaKaRa). (C) Most single mutations in degenerate sites belonged to base transitions and occasional base transversions (e.g. G↔C) (denoted by blue rectangle box); red bar indicates the observed *Bam*HI site mutation.

**Figure 5:**
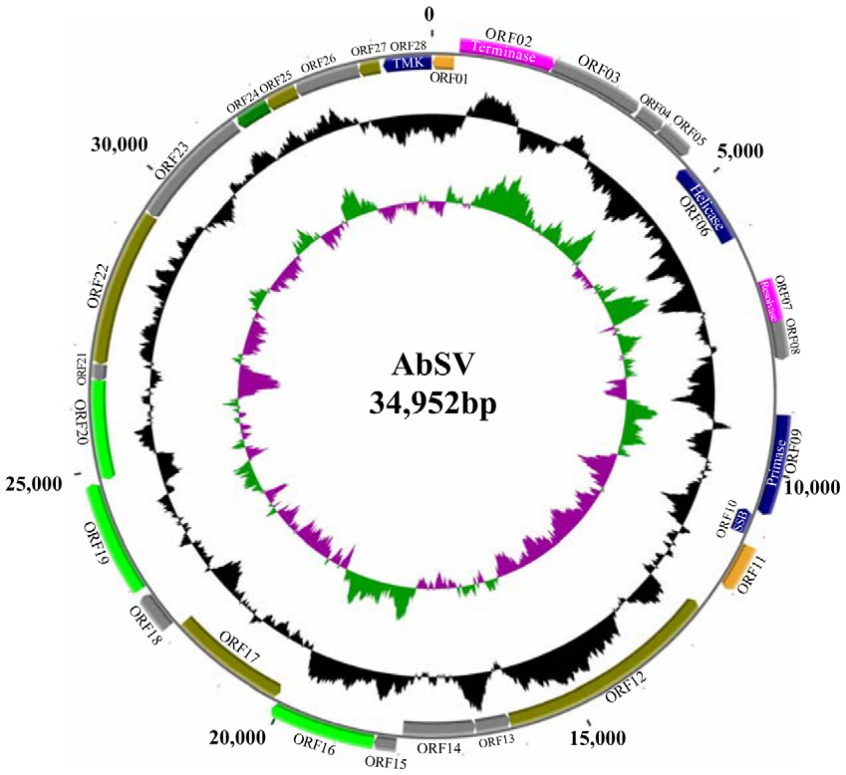
Circular representation of the 34,952-bp AbSV genome. The outside scale is numbered clockwise in base pair (bp). The predicted ORFs are indicated on both strands. Circles 1 and 2 (from outside) are predicted ORFs (forward and reverse strands, respectively), which start with ORF 01 at position 363 bp and are numbered clockwise. ORFs are color-coded according to homologues and putative motif: yellow, most closely related homologue within the GOS data set with a putative function; red, most closely related homologue to bacteria; gray, no significant homologues and unknown function; blue, most closely related homologues to endosymbionts; filemot, having traces of head/tail homology to bacteriophages; light green, sharing detectable homology among AbSV ORFs; dark green, no significant homologues, but containing a conserved domain of unknown function. GC content and GC skew are indicated in the circles 3 and 4, respectively.

AbSV has a double-stranded circular DNA genome of 34, 952 base pairs (GenBank accession no. EU350361). The size and circular structure of the genome were confirmed by restriction enzyme digestions (Figure 4A and 4B) and PCR amplifications. The AbSV genome encodes 28 putative ORFs ranging in size from 91 to 1250 amino acid residues (Figure 5; Table S4). The theoretical coding sequences encompass 94% of the total genomic DNA. Although nucleotide divergences were observed during sequencing, these do not give rise to amino acid residues changes (Figure 4C). The overall amino acid composition of the putative AbSV proteins is strongly biased for residues encoded by A+T rich codons, reflecting the high AT-content (60.5%) of the genome. The positive correlation between AbSV genome size and %GC is in line with a statistical slope inferred by linear regression from over 80 data points from genome sizes of tailed phages and %GC content [22].

Among the predicted AbSV proteins, there are 7 functional proteins (terminase, endonuclease, exonuclease, resolvase, helicase, primase and SSB protein) which are universal proteins of bacteriophages. Also, ORFs 12, 17, 19, 21, 22, 25 and 27 could be traced to the small head/tail subunits of bacteriophages (Table S4). These viral genes coding for DNA packaging proteins, minor head/tail modules (less ten continuous residues homologous to structural protein from tailed phages), and DNA integration and replication, occur together in a linear block on the circular genome, suggesting that AbSV shares not only this common block of genes, but also its gene arrangement with tailed bacteriophages [23], [24]. But, the orientation of AbSV genome structure is the reverse of that found in most T4 or other tailed phages. So, AbSV is referred to as a bacteriophage-related virus.

Despite the bacteriophage-like genomic organization of AbSV, AbSV exhibits only a few of the identified Clusters of Orthologous Groups (COGs) in bacteriophages. However, there was no specific type of bacteriophage COG which was significantly missing in AbSV, except for the lysis category. By this criterion, the absence of a holing or endolysin peptidase may be a distinguishing characteristic between AbSV and other bacteriophages.

Another notable feature of AbSV is the presence of thymidylate kinase, which is a typical gene of vertebrate DNA viruses (e.g. pox and herpes viruses etc.), but not phages with the exception of large phages—myovirus φKZ with larger than 200 kbp [25], [26]. Given the genetic divergence of its multiple bacteriophage-like genes and the presence of a thymidylate kinase, AbSV blurs the frontier between eukaryotic viruses and bacteriophages and is clearly representative of an unknown group of viruses in the marine virosphere. The juxtaposition of these heterogeneous genes in a single genome may be most plausibly explained by multiple horizontal gene transfers (HGTs) between secondary parasites of cellular organisms and bacteriopahges. Thus, AbSV may be a vehicle mediating genetic transfer between hosts and other organisms.

### AbSV genome encompasses a chimeric repertoire of genes of various origins

A large proportion (50%) of the putative ORFs in AbSV remains entirely novel, lacking detectable homology to any known sequences. Among the 14 predicted proteins of AbSV with similarity (e-value smaller than 0.001) to sequences in GenBank and the environmental Global Ocean Survey (GOS) data set, 6 were ORFan proteins which themselves lack any detectable homologs (Table S4). Remarkably, 8 proteins have endosymbiotic, bacteriophagic or bacterial functional homologues (Table S4).

**Figure 6:**
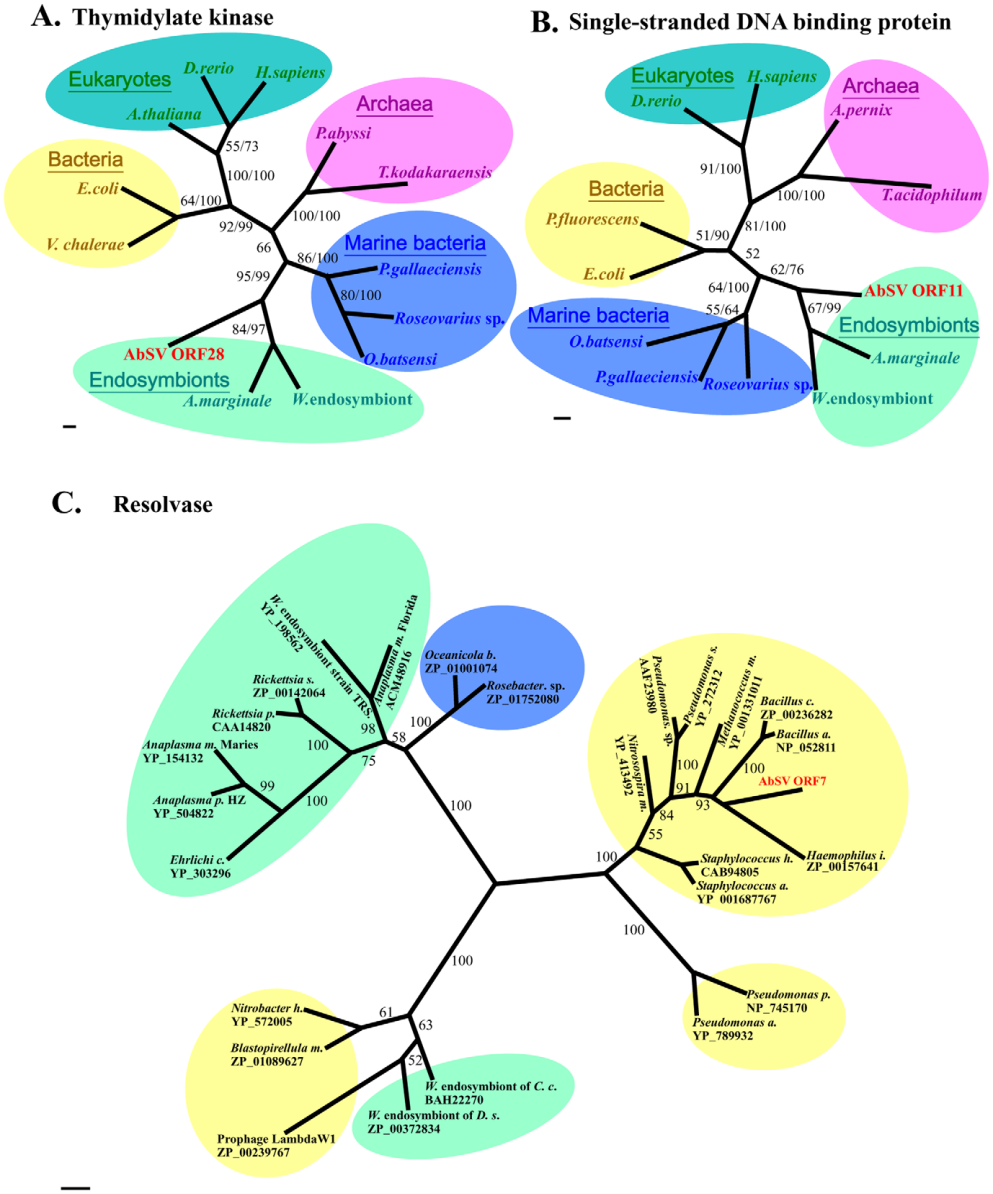
Phylogenetic analyses of AbSV ORFs protein from different domains. Trees were constructed with Mrbayes using the mixed model. Clades with different taxonomic affiliation are colored; ORFs from AbSV are in red. (A) Thymidylate kinase, with homologues in endosymbionts. (B) Single-stranded DNA binding protein and the corresponding AbSV gene of endosymbiont origin. (**C**) Resolvase and the corresponding AbSV gene of bacteria origin. Posterior probabilities are shown along the branches; for trees of thymidylate kinase and SSB protein, maximum likelihood bootstrap values are also indicated. For alignments, see Figure S7 and S9. Scale bars, 0.2 amino acid substitution per site.

First, 4 ORF-encoded products display significant homology to replication-associated components from endosymbionts. Specifically, ORF 6 encodes a protein consisting of two motifs (Walker A and Walker B) involved in viral DNA replication. This protein is a DnaB helicase of P-loop NTPase superfamily that is highly conserved (Figure S9C; Table S4). ORF 9, which is TOPRIM_DnaG_primase gene, encodes a protein containing Zf-CHC2 finger, catalytic core, and nucleotidyl transferase domain, and ORF10 encodes an ssDNA-binding protein (SSB) belonging to RPA2_OBF_family (Table S4). Finally, ORF 28 protein showed significant sequence homology to thymidylate kinase that is highly conserved in different kingdoms (Figure S9A). Phylogenetic analyses of the four universal proteins also revealed that these functional units all cluster with respective endosymbiont-derived homologues (Figure 6A and 6B; Figure S10), suggesting that AbSV could closely connect with intracellular endosymbionts or parasites of AbSV's host organism in DNA duplication processing.

Several putative genes-encoded proteins have significant similarity to bacteriophage or viral proteins. The ORF 1 protein, representing high similarity to a bacteriophage protein, belongs to DUF1064 superfamily which may be a DNA endonuclease (Table S4). The terminase large subunit in AbSV ORF 2, which is uncharacterized by a conserved domain, also appears to be of bacteriophage origin (Figure S11B and 11C; Table S4). Plus, two putative proteins of ORF 12 and 24 are respectively characterized with superfamily PHA01972 similar to structural protein p17 of Enterobacteria phage epsilon 15 and PHA00662 similar to hypothetical protein of *Podoviridae* phage. Thus, these genes (ORFs 1, 2, 12 and 24) seemed to originate in bacteriophages. In contrast, the N-terminal region of ORF19 protein shows limited similarity to an uncharacterized protein in invertebrate iridescent virus 6 (Table S4). This suggests HGTs between the viruses of both prokaryotes and eukaryotes, which can drive the evolution of many phages or viral assemblages [27].

Third, eight AbSV ORF-encoded proteins share considerable similarities with bacterial proteins. The resolvase, whose homologs generally reside in bacteria and phages, is encoded by ORF 7 typically. It might have been obtained by recombinations which partly be attributed to the phylogenetic tree of this protein placing AbSV in the same cluster as bacteria (Figure 6C; Figure S11A). In the same time, the possibility of the so-called bacterial-origin gene as actually integrated phage gene could not be excluded. ORF 11, which is adjacent to an exonuclease VIII gene from bacteria, encodes a protein containing a conserved motif in PRK09709 superfamily (Table S4). ORF16, 20 and 22, which might encode membrane-anchored cell surface proteins, and others (e.g. ORF17 and/or ORF26) are likely involved in multi-drug resistance or virulence, and are most likely derived from bacteria.

Interestingly, insufficient similarities were obtained for the three uncharacterized proteins encoded by ORF16, 19 and 20 (Table S4). This may be the result of sequence resemblance with the adjacent genes. For example, we observed high discrete diversity for the genomic region corresponding to the 5′ terminal of ORF 16 and the 3′ terminal of ORF 17 (ranging from19 kbp to 22.7 kbp positions), and these two regions share a sequence identity of 63.21% (Figure S12).

Therefore, we propose that the genome assemblage of AbSV is composed of genes derived from at least four distinct sources: a new family of viruses (or invertebrate viruses), phages, endosymbionts, and bacteria. It is a chimeric genome, similar to the chimerism reported in the virophage genome [28].

## Discussion

### Specific pathogenecity of AbSV for abalone

In process of AbSV infection, a variety of pathogenic cascades are activated including cellular necrosis and aberrant modification of hemocyanin and ferritin. The abalone hemocyanin is considered as an innate immune molecule [29], based on its similar quaternary structure and immune characteristics to the keyhole limpet hemocyanin, which has been widely used as a hapten carrier, standard antigen, biological response modifier, and immune stimulator [30], [31]. With respect to ferritin, it may act as an innate immune molecule as well [32]. Innate immune responses were triggered passively after AbSV attack, in virtue of abnormal modification of innate immune macromolecules caused by AbSV infection. This response embodies the inequivalent balance between defense and counter-defense among hosts and viruses. The extensive posttranslational modifications (e.g. glycosylation, phosphorylation and protein severing or splicing etc.) occurring in hemocyanin and ferritin and pathogenic relationship between these macromolecules and AbSV pathogenesis remain to be elucidated.

Cellular necrosis during AbSV infection is accompanied by the decomposition of the cytoskeleton (Figure 1A and 1B). The potentially cytoskeleton-derived myosin detected with the hemocyanin functional units in AbSV preparation is reminiscent of the actin cytoskeleton in viral inter-cellular spread. In the cases of vertebrate-infecting viruses, actin-based protrusions were employed for cell-to-cell spread [33]–[35]. It will be worthy to investigate whether AbSV (an invertebrate virus) also employ conventional kinesin-based movement strategies.

### Distinctive sources of functional genes of AbSV

AbSV is characterized by a mosaic genome, with a gene assemblage acquired from a variety of distinct sources. The genes for DNA packaging, and in particular nucleases, appear to be of bacterial or bacteriophage origin, as reported by Boyer et al. [36]; while the genes potentially involved in DNA synthesis and replication are most closely related to homologues in endosymbionts (Figure 6; Figure S10 and S11; Table S4). There seems to be a nonrandom relationship between the functions of the AbSV genes and their inferred origins. It has been hypothesized that the AbSV progenitor was a secondary parasite that shared some replication apparatuses with the same multiplication factory as an archaeal endosymbiont residing in a eukaryotic cell, and HGTs facilitated acquisition of these genes that endowed the recipient with an adaptive advantage from the ingested DNA. Alternatively, those genes which AbSV shares with bacteria may have been acquired through recombination with a phage infecting a bacterium which resided in a eukaryotic cell that also housed a cellular endosymbiont parasitized by pro-AbSV.

### AbSV may lack structural proteins

It is surprising that none of the proteins obtained from virus particle preparation match a putative protein encoded by the AbSV genome at the amino acid sequence level. The AbSV genome has only remnants of several structural protein-encoding genes from phages. The paucity of phage structural genes may partly be accounted for by selection pressures, namely that virus replication appears to be restricted to abalone. Therefore, we hypothesize that the putative genes for viral particle morphogenesis have either lost their ability to be incorporated into AbSV virions, or, more likely, AbSV does not encode its own nucleocapsid proteins. The protein-encoding genes for nucleocapsid of polydnaviruses derivate from nudivirus-related DNA sequences in braconid wasp genome [37], providing a precedence for host-dervied virus capsid proteins.

The denaturation treatment of AbSV preparation primarily indicated that the AbSV genome is not an episome. That is, AbSV may be protected from degradation/truncation by host macromolecules or others because only after denaturation treatment (e.g. SDS treatment) was viral genomic DNA dissociated from pellet suspensions, and the untreated suspension always produced a larger band in gel electrophoresis (Figure S4A).

Ferritin and numerous hemocyanin-like protein units were found in these preparations. Through sucrose gradient ultracentrifugation ferritin was removed from viral suspension along with partial truncation of genomic DNA. Thus, it appears that ferritic ions (or ferritins) play a functional role in maintenance of intactness of the AbSV genomic DNA. Whether ferritic ions resemble other cations (e.g. Na^+^, Mg^2+^ and Co^3+^) which have known ionic effects on the elasticity of phage DNA and packaging motor function [38], remains unclear.

Additionally, most hemocyanin-like particles contain an electron-dense core, as observed via negative staining. These particles, which include long tube particles, may be hollow inside, but do not encompass DNA. Therefore, it seems unlikely that the long tube particles serve as nucleocapsids. However, we can not completely rule out the possibility of a modified hemocyanin subunit complex as a protective protein for AbSV. From a scientific point of view, our results raise several hypotheses. First, the isolated viral DNA may act as a unique form of this virus intermediate. Another hypothesis is that a distinct phage-like plasmid (symbiotic helper), which provides AbSV with a capsid, coexists in abalone. Finally, the heterogeneous DNA may solely represent a vehicle of recombination among the marine organisms. These possibilities need to be confirmed in future studies.

### Perspective on AbSV

From its genome content, AbSV can be described as a viral gene-melting-polymorphic body. Thus far, the AbSV-abalone association represents the only reported case of incorporation of host macromolecular architecture and heterogeneous genetic materials into a virus-like particle that impairs host-cell physiological function. Further investigation for other members of this group of viruses in different abalone species and other mollusks should shed more light on their pathogenicities and also provide new information to probe the viral evolution of several different phylogenetic lineages.

And, AbSV's strategy for adaptive evolution discourages the medical application of phage therapy. As it appears AbSV has done, therapeutic bacteriophage might evolve into pathogenic agent by promiscuous recombination among bacterial pathogens, enteric bacteria and other therapeutic phages. It seems more prudent to the survey potential risks of phage therapy before proceeding.

## Supporting Information

Table S1 Primers used in RT-PCR reaction.
(0.06 MB PDF)

Table S2 Peptide sequences of modified hemocyanin subunits.Footnote: (a)The protein(s) number referred to spot(s) excised from gel shown in Figure 3B. (b)Residue J can be either Ile(I) or Leu(L) and residues B stands for either Gln(Q) or Lys(K). (c)Residues outlined in bold differ from the protein sequence deposited in the NCBInr database. (d)Residues in italic are present in identified protein sequence but defect one residue in peptide segment.
(0.09 MB DOC)

Table S3 Peptide sequences of ferritin subunits isolated from AbSV-infected Halitotis diversicolor.(a)Band protein numbers refer to band excised from gel shown in Supplementary Figure S7A. (b)Residue J can be either Ile(I) or Leu(L) and residues B stands for either Gln(Q) or Lys(K). (c)Residues outlined in bold differ from the protein sequence deposited in the NCBInr database.
(0.03 MB DOC)

Table S4 Properties of the putative ORFs within AbSV genome. Footnote: (a) Data from the GenBank database (http://www.ncbi.nlm.nih.gov/).(b) Data from the Prosite database (http://cn.expasy.org/tools/scanprosite/). (c) Data from the Pfam protein family database (http://pfam.sanger.ac.uk/search). (d) Data from the Superfamily database http://supfam.mrc-lmb.cam.ac.uk/SUPERFAMILY/hmm.html).
(0.13 MB DOC)

Figure S1 Typical clinical sign of sick abalone. Infected abalone H. diversicolor aquatilis with shrunken mantle and pleopod.
(0.57 MB TIF)

Figure S2 Dot/Southern blot hybridization confirmed that AbSV genomic DNA consisted in total DNA from infected abalone. (A) Dot blotting analysis. (B) Southern blotting analysis. Viral DNA and total DNA from infected abalone specifically hybridized to the DIG-labeled probe. No hybridization was detected to DNA from healthy abalone. Positive plasmid control (a and 1), total DNA from artificially infected abalones (b and 5), genomic DNA from healthy abalones (d, 3 and 4), isolated virus from infected abalone (e and 2), and total DNA from naturally infected abalone (f).
(0.05 MB TIF)

Figure S3 Electron microscopy of pathological ultrastructures of AbSV-infected abalone tissues (pleopod and alimentary canal). The myocyte of pleopod displayed diffuse alveolar damage (black arrows) in vertical section (A) and cross section (B) of myofilaments. c and d, In the hepatopancreas cells, the mitochondria cristae appeared to be fragmented (C) and dissolved accompanying marginalization of chromatin and extranuclear aggregates which might originate from fractured pieces (white double arrows) of ERs or other organelles (D). Bars represent 1Î¼m in panel A, 200nm in panel B and C, and 500nm in panel D.
(0.06 MB TIF)

Figure S4 The AbSV genome is encapsided by hemocyanin-like particles. (A) The viral DNA was released from pellet suspension after SDS denaturation. (B) The extracted viral DNA from pellet obtained after PEG-mediated ultracentrifugation (lane 2) was more than one from pellet obtained through sucrose cushion ultracentrifugation (lane 1). The loading volumes were uniform. (C) Each sample lane was divided into two sections (brackets) and analyzed by mass spectrometry. Hemocyanin-like proteins (45 to 100kD region, section 1) act as a major component in pellet obtained after PEG-mediated ultracentrifugation (a); in pellet obtained through sucrose cushion ultracentrifugation (b and c), ferritin subunits (20 to 23 kD region, section 2) have the advantage of amount. (D) Electron microscopy of negatively stained purified particles from PEG-mediated ultracentrifugation. Aggregate distribution of hemocyanin-like particles was present under electron microscopy. Comparatively, the hemocyanin-like particles pellet sedimented by sucrose cushion ultracentrifugation sparsely distributed under electron microscopy (not shown). Note long rectangles (red arrows) like multidecamers, short rectangles (black arrows) which look like tetradecamers, circles (black arrowheads) or cubes (white arrowheads) resembling the typical top-views of or side-views of hemocyanin didecamers, respectively.
(0.38 MB PDF)

Figure S5 The representative MALDI-TOF/TOF MS spectra of trypsin-digested extract of modified hemocyanin subunits. These spectra were shown that identified peptides with [M+H]+ 1055.55, 1342.76, 1335.93, 1532.89 and 1741.12 corresponding to abalone hemocyanin type 1 subunit, and [M+H]+ 1353.79, 1403.85, 1450.87 and 2149.19 to abalone hemocyanin type 2 subunit. D, The peptide (m/z 1615.98) corresponded to myosin. F, The unambiguously identified sequence of peptide with m/z 1215.70 lacked one residue H comparing with corresponding region of abalone hemocyanin type 1 subunit. L, Due to the detectable iminium ions (m/z 84.05 and 101.08) in fragmentation spectrum, it indicated that this peptide contained lysine residue(s). The inferred sequence (WAI/LKPFNR) did not correspond to abalone hemocyanin subunits. Residue J can be either I (Ile) or L (Leu) and residues B stands for either Q (Gln) or K (Lys).
(0.23 MB PDF)

Figure S6 Electron micrograph of native hemocyanin from healty abalone. Not only didecamers (black arrowhead), but also tridecamers (red arrowheads) are observed.
(0.25 MB PDF)

Figure S7 Identification of ferritin by MS. The proteins in viral suspension were denatured and separated on isoelectric focusing (4-7) IPG strips and then separated in the second dimension on a 12.5% SDS-PAGE (A). (B) A representative Q-TOF MS spectrum is shown that identifies spots 1, 2 proteins as the same subunit of abalone ferritin. (C) A representative MALDI-TOF/TOF MS spectrum, which identifies spots 3, 4 proteins as another subunit of ferritin. (D) The AbSV suspension was also separated on non-denaturing gels that was then was subjected to Prussian Blue staining for iron. It suggested that the amounts of iron were incorporated into the viral suspension.
(0.23 MB PDF)

Figure S8 Identification of amino acid sequence modification of ferritin subunit from AbSV-infected H. diversicolor aquatilis. A, The peptide (m/z 1223.8) sequence was be definitely deduced from the typical Q-TOF-MS spectrum. B, The determined sequence has ambiguous homology with corresponding sequence of ferritin subunits of the listed marine species. But the amino acid residues in the corresponding positions of ferrtins within the listed marine species are identical.
(0.17 MB PDF)

Figure S9 Alignment of putative thymidylate kinase (N-terminal) domain, single-strand DNA binding protein (N-terminal) domain, helicase domain (P-loop region) and primase (N-terminal) domain among AbSV and other organisms. The regions with gray background in panel B represent ssDNA binding sites. The identical residues were denoted by asterisks, and conserved or semiconserved residues were denoted by double dots or single dots.
(0.06 MB PDF)

Figure S10 The phylogenetic trees of SSB protein, thymidylate kinase, helicase and primase. ORFs from AbSV are in red. The trees were inferred with neighbor-joining method based on SSB protein and thymidylate kinase (A and B, repectively); sequence data are the same as in Fig. 6b and 6a individually. And trees were constructed with the use of Mrbayes mixed model based on helicase and primase (C and D, respectively). These four genes correspond to origin of endosymbionts. Neighbor joining bootstrap value and posterior probabilities are indicated. Scale bars (C and D), 0.2 amino acid substitution per site.
(0.07 MB PDF)

Figure S11 Alignments of resolvase functional domain and reserved domain of terminase large subunit, and Bayesian tree for terminase large subunit. (A) The regions with gray background corresponds to catalytic residues, yellow background to DNA binding sites. (B) and (C), AbSV is closer to the phage clade in consensus tree of terminase large subunit. Posterior probabilities are shown above each branch. Scale bar, 0.2 amino acid substitution per site.
(0.05 MB PDF)

Figure S12 Comparison of 2.7kb DNA block of AbSV gemome with corresponding sequence in variant, along with alignment of their encoding amino acid sequences. The AbSV variant was referred by AbSV-m19 and its corresponding ORFs were indicated by mORF16 and mORF17. A, The both shared DNA sequence identity of 63.21%. The Variance of DNA sequence did not result in ORFs termination. The ORF17 protein (B) and C-terminal region of ORF16 protein (C) only shared the 57.78% and 67.31% identities with counterparts of variant sequence respectively. But the C-terminal residues, which were denoted by red line, were conserved. The positions of 2.7kb DNA in genome were signed by numbers. The identical bases/residues were highlighted by asterisks.
(0.05 MB PDF)
