# Supplementary material for: A Bacteriophage-Related Chimeric Marine Virus Infecting Abalone
Source: PLoS One. 2010 Nov 5;5(11):e13850. doi: 10.1371/journal.pone.0013850 (PMC2974647; doi:10.1371/journal.pone.0013850)
Supplement: Table S1 — Primers used in RT-PCR reaction. (0.06 MB PDF) [file pone.0013850.s001.pdf]

Table S1. Primers used in RT-PCR reaction.

Used for	Primer name	Sense	Sequence (5’→3’)
RT-PCR	ORF1 F1	Forward	GTATGACTCCCGAGCTGAATATCGAAGAGC
	ORF1 R1	Reverse	CTATTTAACTAATTTAGTATTGTTTGTTG
	ORF3 F1	Forward	ATGGAGTATGAATTCATTGATTATTTAC
	ORF3 R1	Reverse	CAATACCATCTTCTGTTTCAAACGACA
	ORF9 F1	Forward	ATGGAGACATTAATGACCCAAGCTTCAAG
	ORF9 F1	Reverse	TCGATTTGATGGGTAGTTGTACTAGTCC
	ORF11 F1	Forward	ATGAAAAACAGTATTAACAAAACTATAT
	ORF11 R1	Reverse	TTAAAACGGCACTTCATCATCGTCTTCAG
	ORF15 F1	Forward	CAGGGCATTGGATGCGGCTATTGCCGGG
	ORF15 R1	Reverse	TTAATGCCTCAGCATTGCCTTGATATGAT
	ORF17 F1	Forward	ATGAGGGCATTGGGCAATGCTTTGGGTATT
	ORF17 R1	Reverse	TTAACACTACCCCTAGCTCCAATAGTACC
	ORF20 F1	Forward	ATGGAATATCTAGTTAAAAGAAAATTACTG
	ORF20 R1	Reverse	ACCCCCGAGAATCATTCCAATTGAAATTC
	ORF21 F2	Forward	GGGCGGATGAGCTAACTGGTGATCCAAAC
	ORF21 R2	Reverse	CTACCTTTTGTAAAACCTGTATATACAAT
	ORF23 F1	Forward	GTACTATTGCTGTAGAGGATATCACGGCAG
	ORF23 R1	Reverse	AGCTTTTATTAACTAGTATATATCTTAACAG
	ORF24 F1	Forward	ATGAATTCAATGCTACGAGTGACCACCTTATC
	ORF24 R1	Reverse	CTACAGTAAATCATTTCCTAATGACTGGAC
