# Supplementary material for: A Bacteriophage-Related Chimeric Marine Virus Infecting Abalone
Source: PLoS One. 2010 Nov 5;5(11):e13850. doi: 10.1371/journal.pone.0013850 (PMC2974647; doi:10.1371/journal.pone.0013850)
Supplement: Table S2 — Peptide sequences of modified hemocyanin subunits.Footnote: (a)The protein(s) number referred to spot(s) excised from gel shown in Figure 3B. (b)Residue J can be either Ile(I) or Leu(L) and residues B stands for either Gln(Q) or Lys(K). (c)Residues outlined in bold differ from the protein sequence deposited in the NCBInr database. (d)Residues in italic are present in identified protein sequence but defect one residue in peptide segment. (0.09 MB DOC) [file pone.0013850.s002.doc]

**Table S2. Peptide sequences of modified hemocyanin subunits**

| Proteins number^a^ (molecular weight) | | Peptide segment (M+H) ^+^ mass | De novo sequencing^b,c,d^ | The matching sequence identified by MASCOT search ^e^  Blast analysis & homologous peptide description ^f^ | hemocyanin FUs and/or other proteins in spot protein(s) |
| --- | --- | --- | --- | --- | --- |
| 1  (110kD) | | 933.58  1031.58  1307.69  1353.79  1403.87  1397.74  1724.88  1863.01 | JFAJWBR  WAJBPFNR  AFHFDVTDAER  JYTVBFBDAJR  JFVTBVEDAJJR  FGYHYDNJNVR  HNPFHD**AD**VAF**B**NGR  SVDGYBATVEYHGJPAR | ^129^IFAIWQR^135^ Hemocyanin 2 [*H. tuberculata*]^e^ _CAC82192.1  ^2093^WAIKPFN^2099^ capsid protein [*Acanthamoeba polyphaga* mimivirus]^f^ _YP_142794.1; ^55^ALQPFNR^61^ hypothetical protein gp019 [*Pseudomonas* phage EL] ^f^ _YP_418052.1  ^270^AFHFDVTDAER^284^ Hemocyanin 2 [*H. tuberculata*]^e^  ^1650^LYTVQFQDALR^1660^ Hemocyanin 2 [*H. tuberculata*]^e^  ^406^LFVTQVEDALIR^417^ Hemocyanin 2 [*H. tuberculata*]^e^  ^3089^FGYHYDNLNVR^3099^ Hemocyanin 2 [*H. tuberculata*]^e^  ^455^HNPFHDVEVAFENGR^469^ Hemocyanin 2 [*H. tuberculata*]^f^; ^251^NFPASVQFQNGR^262^ Capsid protein [Bovine enteric virus]^f^_AAT28379.1;  ^364^SVDGYQATVEYHGLPAR^380^ Hemocyanin 2 [*H. tuberculata*]^e^ | hemocyanin 2 FU-a, -b, -e, -h |
| 2  (100kD) | | 1055.55  1211.75  1342.73  1507.73  1532.89 | YDNPPFFR  JJALBAENAJR  JWAJWBEJBR  EHAIPFDVFNYR  JYV**T**BVENAJJBR | ^464^YDNPPFFR^471^ Hemocyanin 1 [*H. tuberculata*]^f^_CAC20588.1  ^2^LLALQAENALR^12^ Hemocyanin 1 [*H. tuberculata*]^e^  ^193^EHAIPFDVFNYR^204^ Hemocyanin 1 [*H. tuberculata*]^e^  ^557^LWAIWQELQR^566^ Hemocyanin 1 [*H. tuberculata*]^e^  ^1337^LYVVQVENALLNR^1349^ Hemocyanin 1 [*H. tuberculata*]^f^ | hemocyanin 1 FU-a, -b, -d |
| 3, 4  (80Kd, 70Kd) | | 942.52  1101.67  1382.81  1615.98  1625.96  1942.18  1983.17 | GYSAEFJR  ANAEVQQWR  RFFPNPDFTQQ  AQJEEAQDQJESVR  TNSYPNJVFDHYR  …EESTJQHESQVASJR  …EJHDJTDQJSEGGR | ^29^GYSAEYLR^36^ ORF100 [*Pseudomona*s phage phiKZ]_NP_803666.1  ^1363^ANAEVQQWR^1371^ myosin heavy chain [*Loligo pealei*]^e^_AAC24207.1  ^1278^RFFPNPDFTQQ^1288^ Hemocyanin 2 [*H. tuberculata*]^f^  ^1479^AQIEESQEQLESVK^1490^ myosin heavy chain [*Dugesia japonica*]^f^_BAA34954.1  ^73^AQLEEAHDQIESVK^80^ myosin heavy chain [ *Fasciola hepatica*]^f^_CAC86158.1  ^116^TNSYPNJVFDHYR^128^ Hemocyanin [*H. diversicolor supertexta*]_ACF70600.1; NSFPSILFDHYR^3191^ Hemocyanin 1 [*H. tuberculata*]^f^  ^1178^EEATMQHESQIATLR^1192^ myosin heavy chain [*Loligo pealei*]^e^_AAC24207.1  ^1505^EIHDLTDQLSEGGR^1518^ myosin heavy chain [*Placopecten magellanicus*]^e^_AAB03661.1 | Myosin; hemocyanin 2 FU-d; hemocyanin 1 FU -h |
| 5  (60kD) | | 1027.57  1179.72  1215.67  1233.59  1625. 95  2192.30  1741.12 | JHTJBMER  JFAGFVJSGJR  APTJEFEPG*VR*  FNYEYDNJR  TNSYPNJVFDHYR  APJHPFNYE**N**VNEDEFTR  GBDJBDJEVVJNEJR | ^2997^LHTIQMER^3004^ Hemocyanin 1 [*H. tuberculata*]^e^  ^3222^IFAGFVLSGLR^3232^ Hemocyanin 1 [*H. tuberculata*]^e^  ^2900^APTIEFEPGVHR^2911^ Hemocyanin 1 [*H. tuberculata*]^f^  ^129^FNYEYDNLR^137^ Hemocyanin [*H. diversicolor supertexta*]^e^; ^3192^FNYEYDNMR^3200^ Hemocyanin 1 [*H. tuberculata*]^f^  ^116^TNSYPNJVFDHYR^128^ Hemocyanin [*H. diversicolor supertexta*], ^3180^ NSFPSILFDHYR^3191^ Hemocyanin 1 [*H. tuberculata*]^f^  ^3162^APLHPFNYETVNEDEFTR^3178^ Hemocyanin 1 [*H. tuberculata*]^f^  ^140^GQDIQDLEVVLNELR^154^ Hemocyanin [*H.s diversicolor supertexta*]; ^3203^GQDIHELEEVIQELR^3217^ Hemocyanin 1 [*H. tuberculata*]^f^ | hemocyanin 1 FU -h |
| 6  57kD | | 933.58  1031.57  1101.56 | JFAJWBR  WAJBPFNR  ABTNYWYR | ^231^LFAIWQR^237^ Hemocyanin 1 [*H. tuberculata*]^e^  ^2093^WAIKPFN^2099^ capsid protein [Acanthamoeba polyphaga mimivirus]^f^ _YP_142794.1; ^55^ALQPFNR^61^ hypothetical protein gp019 [Pseudomonas phage EL] ^f^ _YP_418052.1  ^133^AQTNYWYR^140^ Hemocyanin 1 [*H. tuberculata*]^e^ | hemocyanin 1 FU-a |
| 7  52kD | | 933.59  1342.80  1353.80 | JFAJWBR  JWAJWBEJBR  JYTVBFBDAJR | ^129^IFAIWQR^135^ Hemocyanin 2 [*H. tuberculata*]^e^  ^1382^LWAIWQELQR^1373^ Hemocyanin 2 [*H. tuberculata*]^e^  ^1650^LYTVQFQDALR^1660^ Hemocyanin 2 [*H. tuberculata*]^e^ | hemocyanin 2 FU-a, -d, -e |
| 8  49kD | | 1215.75  1576.93  2107.04  2149.15  2185.23 | APTJEFEPG*VR*  JWA**J**WQAJQEYR  DMJFNDPEHGSES**YFF**R  YEYDTJDFNGJSJSQJDR  …GJTDEJNPDDETR | ^2900^APTIEFEPGVHR^2911^ Hemocyanin 1 [*H. tuberculata*]^e^  ^2724^IWAVWAQLQEYR^2735^ Hemocyanin 1 [*H. tuberculata*]^e^  ^2649^DMLFNDPEHGSESFFYR^2665^ Hemocyanin 1 [*H. tuberculata*]^f^  ^1025^YEYDTLDFNGLSISQIDR^1042^ Hemocyanin 2 [*H. tuberculata*]^f^  ^996^GJTDEJNPDDETR^1008^ Hemocyanin 2 [*H. tuberculata*]^f^ | hemocyanin 1 FU-g; hemocyanin 2 FU-c |
| 47kD | 9 | 938.66  1215.70  1335.95  1576.97  2046.05  2107.12  1951.19  1965.22 | TFAAFJJR  APTJEFEPG*VR*  JFAAFJJSGJBR  IWA**J**WQAJQEYR  DMJFNDPEHGSES**YFF**R  …SD**E**JNHNP…  TPJRPFSD**E**JNHNPVTK | ^2707^TFAAFLLR^2714^ Hemocyanin 2 [*H. tuberculata*]  ^2900^APTIEFEPGVHR^2911^ Hemocyanin 1 [*H. tuberculata*]^e^  ^2811^IFAAFLLSGIKR^2822^ Hemocyanin 1 [*H. tuberculata*]^f^  ^2724^IWAVWQALQEYR^2735^ Hemocyanin 1 [*H. tuberculata*]^f^  ^2649^DMLFNDPEHGSESFFYR^2665^ Hemocyanin 1 [*H. tuberculata*]^e^  ^2649^DMLFNDPEHGSESFFYR^2665^ Hemocyanin 1 [*H. tuberculata*]^e^  ^2757^SDDINHNP^2764^ Hemocyanin 1 [*H. tuberculata*]^f^  ^2751^TPLRPFSDDINHNPVTK^2767^ Hemocyanin 1 [*H. tuberculata*]^f^ | hemocyanin 1 FU-g; hemocyanin 2 FU-g |
|  | 10 | 1055.55  1342.76  1215.70  1552.96  1382.79  1560.04  1651.96 | YDNPPFFR  JWAJWBEJBR  APTJEFEPG*VR*  AN**A**BPJDVFEYNR  RFFPNPDF**S**BB  JYVJBVENAJJER  YDNJ**JY**NHFSJPR | ^464^YDNPPFFR^471^ Hemocyanin 1 [*H. tuberculata*]^f^  ^193^EHAIPFDVFNYR^204^ Hemocyanin 1 [*H. tuberculata*]^e^  ^2900^APTIEFEPGVHR^2911^ Hemocyanin 1 [*H. tuberculata*]^e^  ^278^ANSKDVFEYNR^2780^ Hemocyanin 1 [*H. tuberculata*]^f^  ^1278^RFFPNPDFTQQ^1288^ Hemocyanin 2 [*H. tuberculata*]^f^  ^1234^LYVLQVENALLER^1246^ Hemocyanin 2 [*H. tuberculata*]^f^  ^1436^YDNLEFNHFSIPR^1448^ Hemocyanin 2 [*H. tuberculata*]^e^ | hemocyanin 1 FU-b, -g;  hemocyanin 1 FU-d |
| 45kD | 11 | 1213.74  1658.97  1904.06  2149.19  2185.29 | JJTVBGENAJR  TAGGFBBJAAFHGEPK  PNWYSGHJDSVGVDTTR  YEYDTJDFNGJSJSQJDR  KPJBPFGJTDEJNPDDETR | ^818^LLTVQGENALR ^828^ Hemocyanin 2 [*H. tuberculata*]^e^  ^776^TAGGFQQIAAFHGEPK^791^ Hemocyanin 2 [*H. tuberculata*]^e^  ^870^PNWYSGHIDSVGVDTTR^884^ Hemocyanin 2 [*H. tuberculata*]^e^  ^1025^YEYDTLDFNGLSISQIDR^1042^ Hemocyanin 2 [*H. tuberculata*]^e^  ^990^KPLQPFGLTDEINPDDETR^1008^ Hemocyanin 2 [*H. tuberculata*]^e^ | hemocyanin 2 FU-b, -c |
|  | 12 | 938.63  1215.74  1323.78  1450.87  2037.03  2098.09 | TFAAFJJR  APTJEFEPG*VR*  SPTJEH**D**JGA**B**R  SIPELHDLLEER  ... SESFFYR  …F**P**DPEBGSESFFYR | ^2707^TFAAFLLR^2714^ Hemocyanin 2 [*H. tuberculata*]  ^2900^APTIEFEPGVHR^2911^ Hemocyanin 1 [*H. tuberculata*]^e^  ^2797^SPTIEHELGAHR^2807^ Hemocyanin 2 [*H. tuberculata*]^e^  ^2690^SIPELNDLLEER^2701^ Hemocyanin 2 [*H. tuberculata*]^e^  ^2555^SESFFYR^2561^ Hemocyanin 2 [*H. tuberculata*]^e^  ^2448^FNDPEQGSESFFYR^2561^ Hemocyanin 2 [*H. tuberculata*]^e^ | hemocyanin 2 FU-g;  hemocyanin 1 FU-g |

Footnote: ^a^The protein(s) number referred to spot(s) excised from gel shown in Figure 3B.

^b^Residue J can be either Ile(I) or Leu(L) and residues B stands for either Gln(Q) or Lys(K).

^c^Residues outlined in bold differ from the protein sequence deposited in the NCBInr database.

^d^Residues in italic are present in identified protein sequence but defect one residue in peptide segment
