# Supplementary material for: A Bacteriophage-Related Chimeric Marine Virus Infecting Abalone
Source: PLoS One. 2010 Nov 5;5(11):e13850. doi: 10.1371/journal.pone.0013850 (PMC2974647; doi:10.1371/journal.pone.0013850)
Supplement: Table S3 — Peptide sequences of ferritin subunits isolated from AbSV-infected Halitotis diversicolor.(a)Band protein numbers refer to band excised from gel shown in Supplementary Figure S7A. (b)Residue J can be either Ile(I) or Leu(L) and residues B stands for either Gln(Q) or Lys(K). (c)Residues outlined in bold differ from the protein sequence deposited in the NCBInr database. (0.03 MB DOC) [file pone.0013850.s003.doc]

***Table S3. Peptide sequences of ferritin subunits isolated from AbSV-infected Halitotis diversicolor***

| Band or spot proteins^a^ (molecular weight) | Peptide segment (M+H) ^+^ mass | De novo sequencing^b,c^ | The matching sequence identified by MASCOT analysis^e^  Blast analysis & homologous peptide Peptide description^f^ | Deduction of ferritin subunit |
| --- | --- | --- | --- | --- |
| 1,2  (23kD) | 1026.61  1329.72  1748.87 | JSDHVTNJK  SVNBEFJDJHK  VGPGJGEYBFDHET**H**S | ^151^LSDHVTNLK^159^ ferritin subunit [*H. discus hannai*]^e^  ^107^SVNQEFLDLHK^117^ ferritin subunit [*H. discus hannai*]^e^  ^156^VGPSLGEYQFEHETLS^171^ ferritin subunit [*H. discus hannai*]^f^ | Ferritin heavy chain subunit |
| 3,4  (20kD) | 828.57  1500.81  1592.98  1731.89 | JVJBDJK  BNFHVESEAGJNR  JJDJHAVASK  TYBSJAFYFDR | ^78^IVLQDIK^84^  ferritin subunit [*H. diversicolor*]^e^  ^8^QNFHVESEAGINR^20^ ferritin subunit [*H. diversicolor*]^e^  ^112^LLDLHAVASK^121^ ferritin subunit [*H. diversicolor*]^e^  ^31^TYQSIAFYFDR^41^ ferritin subunit [*H. diversicolor*]^e^ | Ferritin light chain subunit |

^a^Band protein numbers refer to band excised from gel shown in Supplementary Fig. S7A.

^b^Residue J can be either Ile(I) or Leu(L) and residues B stands for either Gln(Q) or Lys(K).

^c^Residues outlined in bold differ from the protein sequence deposited in the NCBInr database.
