# Supplementary material for: A Bacteriophage-Related Chimeric Marine Virus Infecting Abalone
Source: PLoS One. 2010 Nov 5;5(11):e13850. doi: 10.1371/journal.pone.0013850 (PMC2974647; doi:10.1371/journal.pone.0013850)
Supplement: Table S4 — Properties of the putative ORFs within AbSV genome. Footnote: (a) Data from the GenBank database (http://www.ncbi.nlm.nih.gov/).(b) Data from the Prosite database (http://cn.expasy.org/tools/scanprosite/). (c) Data from the Pfam protein family database (http://pfam.sanger.ac.uk/search). (d) Data from the Superfamily database http://supfam.mrc-lmb.cam.ac.uk/SUPERFAMILY/hmm.html). (0.13 MB DOC) [file pone.0013850.s004.doc]

**Table S4. Properties of the putative ORFs within AbSV genome.**

| ORFs | Nucleotide position  (length [aa]) | Molecular Mass (KDa) | The closest Match^a^ | | | | Conserved domain or signature^b,c,d^  (CD/Prosite accession no.) | Putative function of encoded peptide |
| --- | --- | --- | --- | --- | --- | --- | --- | --- |
|  |  |  | BlastP  Score | %  Identity | Accession  No. | Species |  |  |
| ORF1 | 1<—363 (120) | 13.93 | 6e-16  70.9 (3e-11) | 47(42/88)  42 (43/101) | GOS_7688360  YP_001686850 | Azospirillum phage Cd (Phage protein of unkown function) | PF06356 DUF1064 Protein of unknown function (1-98, 3e-12, It may be endonucleases which function in phage genome segregation, or the repair of double-stranded breaks introduced during either this process or DNA replication.) | — |
| ORF2 | 439—>1962 (507) | 57.99 | 3e-24  94.7 (2e-17)  84 (3e-14) | 25(127/501)  22 (114/496)  22 (107/478) | GOS_1271718  YP_001667985  YP_002290878 | Pseudomonas putida GB-1(putative phage terminase, large subunit)  Clostridium phage phiCD27 (putative terminase B large subunit ) | PF04851 ResⅢ (45-90, 0.39, binding DNA and hydrolase activity)  PF03237 Terminase_6 (77-484, 0.79, Terminase in a group of plasmid encodes proteins specifically found in *Borrelia* genus)  Pfam-B 93161 (77-284, 8e-06, Terminase DNA-binding hydrolase DNA large pacase packaging ATP-binding nuclease) | Terminase |
| ORF3 | 1952—>3460 (502) | 56.45 | 40 (0.42)  36.6 (4.7) | 24 (42/174)  26 (40/153) | YP_560995  ZP_02950398 | Burkholderia xenovorans LB400 (RND efflux system, outer membrane lipoprotein, NodT family)  Clostridium butyricum 5521 (3-dehydroquinate dehydratase, type I) | PF00004 AAA ATPase (387-438, 0.14, ATP binding protein often perform chaperone-like function that assist assembly, operation, or disassamebly of protein complex)  ABC transporter ATPase domain-like (16-487, 0.082) Famliy | — |
| ORF4 | 3494—>3937 (147) | 17.25 | 35 (1.6) | 34 (17/49) | YP_001001667 | Halorhodospira halophila SL1 (Phenylacetate--CoA ligase ) | — | — |
| ORF5 | 3937—>4518 (193) | 21.48 | 36.2 (1.2)  35 (2.7) | 27（28/101）  30 (24/79) | NP_044564  ZP_02688150 | Toxoplasma gondii RH ( Clp protease ATP binding subunit)  Listeria monocytogenes FSL J2-071 (NAD-dependent DNA ligase LigA ) | Periplasmic binding protein-like II (82-151, 3.38e-02) superfamily | — |
| ORF6 | 4621<—6063 (480) | 55.74 | 178 (7e-43)  177 (2e-43)  2e-37 | 27 (131/481)  29 (143/478)  32(96/296) | YP_154034  YP_198130  GOS_4473 | Anaplasma marginale str. St. Maries ( replicative DNA helicase )  .Wolbachia endosymbiont strain TRS of Brugia malayi (replicative DNA helicase ) | ABC_ATPase superfamily DnaB domain  PF03796 DnaB-like helicase C terminal domain (180-394, 2.6e-14) | replicative DNA helicase |
| ORF7 | 6872—>7573 (233) | 26.67 | 115 (2e-24)  115 (3e-24)  2e-22 | 37 (68/181)  37 (67/181)  32(60/183) | AAF23988  YP_272312  GOS_1724577 | Pseudomonas sp. TW3 (putative transposase)  Pseudomonas syringae pv. phaseolicola 1448A (putative resolvase ) | PF00239 Resolvase, N terminal domain (DNA Recombinase), PF00466 Ribosomal L10 (48-135, 0.61, ribosome biogenesis and assembly), PF00611 Fes/CIP4 homology domain (90-182, 0.076, It may mediate cytoskeletal rearrangements required for cytokinesis.)  PS00398 Recombinases_2  Ser_Recombinase superfamily (12-145, 6.23e-32) | DNA Resolvase |
| ORF8 | 7491—>8177 (228) | 26.00 | 40.4 (0.093) | 22 (34/150) | YP_171810 | Synechococcus elongatus PCC 6301 (ATPase) | PF08537 Fungal Nap binding protein NBP1 (45-64, 0.56, It may be essential for the G2/M transition of the cell cycle.) | — |
| ORF9 | 9048—>10676 (542) | 62.32 | 191(2e-46)  192(6e-47)  2e-45 | 32 (131/405)  34(124/364)  30(113/370) | YP_198027.1  YP_605374.1  GOS_11117669 | Wolbachia endosymbiont strain TRS of Brugia malayi  Deinococcus geothermalis DSM 11300 | zinc beta-ribbon (6-95, 3.48e-28) ; DNA Primase core (105-350, 1.38e-46) superfamily  CHC2 zinc finger (2-96, 3.7e-30, DNA-binding in DNA primase); DNA primase catalytic core, N-terminal domain (108-227, 4.2e-27, catalyse the relaxation of DNA supercoiling by causing transient double strand breaks); Toprim domain (233-307, 5.6e-08, DNA primase is a nucleotidyltransferase which synthesizes the oligoribonucleotide primers required for DNA replication on the lagging strand of the replication fork.)  Toprim_N superfamily, DnaD domain | DNA primase |
| ORF10 | 10673<—11077 (134) | 15.12 | 153 (4e-36)  151 (1e-35)  4e-36 | 53 (70/131)  55 (60/122)  59(68/115) | EDT29906  YP_505066  GOS_9331480 | Oligotropha carboxidovorans OM5 (single-strand binding protein)  Anaplasma phagocytophilum HZ (single-strand binding protein ) | PF00436 Single-strand binding protein family (5-114, 1.4e-24)  Single strand DNA-binding domain, SSB (1-134, 6.10e-05) | single-stranded DNA-binding protein (SSB) |
| ORF11 | 11212—>11988 (258) | 29.64 | 2e-07  49.3 (3e-04) | 25(59/235)  29 (33/112) | GOS_8588039  YP_880103 | Mycobacterium avium 104 (gp60 protein) | PRK09709 Superfamily (658-872, 0.002)  MAC/Perforin domain (83-98, 0.46, These channels disrupt the phospholipid bilayer of target cells, leading to cell lysis and death.); RasGAP C-terminus (163-188, 0.24, small GTPase mediated signal transduction) | exonuclease VIII |
| ORF12 | 12427<—16176 (1250) | 135.87 | 50.4 (9e-04)  41.2 (0.53)  40.8 (0.70)  38.5 (3.5) | 19 (124/629)  24 (38/154)  23 (74/313)  21 (92/427) | YP_024981  ZP_02154026  YP_001809972  ABW80568 | Vibrio phage VP5 (gP18)  Oceanibulbus indolifex HEL-45 (major capsid protein, HK97 family )  Burkholderia ambifaria MC40-6 (filamentous haemagglutinin family outer membrane protein)  Euagrus chisoseus ( fibroin 1 ) | PHA01972 superfamily (214-806, 0.002)  DUF1228 protein of function unkown (135-210, 0.28, It represents the N terminus of several putative bacterial membrane proteins, which may be sugar transporters.); PF03087 Arabidopsis protein of unknown function (501-527, 0.95); Ribbon-helix-helix protein, CopG family (538-585, 0.91, CopG, is responsible for the regulation of plasmid copy number.); Bunyavirus nucleocapsid (N) protein (922-932, 0.85); TAFII55 protein conserved region (1056-1069, 0.24, it participates in the assembly of the transcription preinitiation complex, regulation of transcription.)  GroEL equatorial domain-like (110-985, 5.58e-12), P-loop containing nucleotide triphosphate hydrolases (20-1182, 1.42e-01), Thiamin diphosphate-binding fold (THDP-binding) (87-1125, 2.0e-01, 0.040) | — |
| ORF13 | 16173<—16754 (193) | 21.04 | 35.4 | 27(27/98) | YP_330739 | Natronomonas pharaonis DSM 2160 (transmembrane protein, homolog 2 to type II secretion system proteins TadC) | NAD(P)-binding Rossmann-fold domains (94-164,1.16e-02)  Ribonuclease H-like (42-129, 1.56e-01) superfamily DnaQ-like 3'-5' exonuclease family | — |
| ORF14* | 16754<—17962 (402) | 44.23 | 44.3  42.7 (0.048)  42.7 (0.048) | 21 (62/286)  20 (44.219)  19 (45/227) | ZP_02748195  YP_001008743  ABE11470 | Clostridium difficile QCD-63q42 (putative surface protein)  Prochlorococcus marinus str. AS9601 (carboxyl-terminal protease)  uncultured Prochlorococcus marinus clone HOT0M-7C8 (PDZ domain protein ) | PF02637 GatB_Yqey family, GatB domain (38-167, 0.49, Yqey and its relatives have a role in tRNA metabolism.)  DUF1274 protein of unknown function (56-75, 0.4, this family consists of several Chordopoxvirus proteins of around 160 residues in length.)  P loop containing nucleoside triphosphate hydrolases (1-368, 2.15e-01) | — |
| ORF15 | 18048—>18410 (120) | 13.13 | 32.7 (7.7) | 32.7 (17/49) | YP_349527 | Pseudomonas fluorescens PfO-1 (TonB-dependent siderophore receptor ) | — | — |
| ORF16 | 18412—>20112 (566) | 37.79 | 48.5 (8e-04) | 25 (51/202) | YP_111446 | Burkholderia pseudomallei K96243 (membrane-anchored cell surface protein) | Elongation factor P, C-terminal (161-209, 0.97) | — |
| ORF17* | 20104<—22092 (662) | 72.77 | 50.8 (3e-04)  46.6 (0.006)  37.7 (2.9) | 25 (73/282)  27 (41/151)  25 (29/114) | YP_664368  XP_646815  YP_001429621 | Helicobacter acinonychis str. Sheeba (putative vacuolating cytotoxin (VacA) paralog)  Dictyostelium discoideum AX4 (cyclin-like F-box containing protein)  Bacillus phage 0305phi8-36 (virion structural protein ) | NAD(P)-binding Rossmann-fold domains superfamily  Asparaginase (54-305, 0.53) | — |
| ORF18 | 22207—>22833 (208) | 24.94 | 38.9 (0.21)  34.3(5.3) | 25 (35/137)  30 (21/69) | XP_680202  YP_001376608 | Plasmodium berghei strain ANKA (hypothetical protein)  Bacillus cereus subsp. cytotoxis NVH 391-98 (nitrate reductase molybdenum cofactor assembly chaperone) | — | — |
| ORF19* | 22942—>24792 (616) | 66.60 | 53.5 (5e-05)  43.9 (0.038) | 27(56/207)  29 (51/174 ) | NP_149906  YP_555132 | Invertebrate iridescent virus 6 (443R)  Burkholderia xenovorans LB400 (Adhesin HecA ) | PF04206 Tetrahydromethanopterin S-methyltransferase, subunit E (27-220, 0.48, using a transmethylation reaction to drive an energy-conserving sodium ion pump); DUF1675 (RNA-binding protein); PF02924 HDPD (Bacteriophage lamda head decoration protein D) (102-109, 0.038); Phage T7 capsid assembly protein (325-337, 0.5) | — |
| ORF20 | 24795<—26462 (555) | 58.65 | 49.7 (6e-04)  48.9 (0.001) | 35（152/425）  23 (64/278) | EAR61561.1  ZP_02889734 | Oceanospirillum sp. (lipoprotein receptor-related protein)  Burkholderia ambifaria IOP40-10 (filamentous haemagglutinin family outer membrane protein ) | Trans glycosidases (22-79, 2.95e-01) superfamily | — |
| ORF21* | 26730—>26455 (91) | 10.20 | 43.5 (0.012)  40.5 (0.095)  35.4 (3.2)  32.9 (19)  32.9 (19) | 43 (19/44)  41 (22/53)  63 (12/19)  46 (14/30)  38 (26/67) | XP_001442560  NP_275981  NP_046904  YP_002412171  YP_001384742 | Paramecium tetraurelia strain d4-2 (hypothetical protein)  Methanothermobacter thermautotrophicus (glycerol phosphate cytidylyltransferase )  Enterobacteria phage N15 (gp9 )  Escherichia coli UMN026 (DNA packaging protein from bacteriophage origin)  Clostridium botulinum A str. ATCC 19397 (TP901 family phage tail tape measure protein) | PF04740 Transposase_30 (38-65, 0.97, this family of putative transposases includes mostly Bacillus members. However, we have also found a Bacillus subtilis bacteriophage SPbetac2 homologue, possibly arising as aresult of horizontal transfer) | — |
| ORF22* | 26732<—29380 (822) | 93.85 | 65.1 (2e-08)  62.4 (2e-07)  59.7 (1e-06)  58.2 (3e-06)  56.2 (1e-05) | 27 (67/241)  29 (63/215)  22 (87/390)  31(49/156)  23 (72/312) | YP_001818573  ZP_01054714  YP_311286  XP_811301  ZP_01444683 | Opitutus terrae PB90-1 (cell wall surface anchor family protein)  Roseobacter sp. MED193 (type I secretion target repeat protein)  Shigella sonnei Ss046 (prophage tail fibre proteins)  Trypanosoma cruzi strain CL Brener (mucin-associated surface protein (MASP), putative )  Roseovarius sp. HTCC2601 (tail fiber protein, putative) | PF06519 TolA (33-391, 0.38, being involved in the translocation of group A colicins); DUF15420 (205-263, 0.73, cell surface protein involved in antibiotic resistance or cellular adhension); PF02946 GTF2I-like repeat (427-436, 0.81, DNA binding protein).  Composite domain of metallo-dependent hydrolases (7-8814, 93e-01) | — |
| ORF23 | 29384<—31468 (737) | 81.32 | 41.2 (0.31)  40.4 (0.52) | 25 (41/158)  21(90/410) | XP_001610140  YP_001273970 | Babesia bovis (hypothetical protein)  Methanobrevibacter smithii ATCC 35061 (adhesin-like protein ) | PF05317, Thermopsin (132-365, 0.71); PF02409 O-methyltransferase N-terminus (541-630, 0.56) | — |
| ORF24 | 31506<—32091(194) | 22.21 | 42.0(0.024) | 24 (42/173) | ZP_03464580 | Desulfovibrio salexigens DSM 2638 (hypothetical protein) | PHA00662 superfamily (38.75, 8e-04) | — |
| ORF25* | 32090<—32617 (175) | 18.68 | 38.1 (0.24)  35.0 (2.0) | 30 (32/106)  23 (43/186) | ZP_00517493  YP_001847376 | Crocosphaera watsonii WH 8501 (Acetate kinase)  Acinetobacter baumannii ACICU (Phage-related protein tail component) | Bacterial adhesins superfamily (3.0e-01); Dr-family adhesin (Pfam 046190). | — |
| ORF26 | 32617<—33717 (366) | 40.12 | 39.7 (0.36)  38.1 (1.0) | 28 (46/162)  21 (43/221) | YP_744301  AAA45470 | Granulibacter bethesdensis CGDNIH1 (multidrug resistance ABC transporter ATP-binding and permease Protein)  Hepatitis A Virus( polyprotein precursor， it may be drug-binding pocket) | PF09024 Sak_Polo box (289-300, 0.15, regulation of cell cycle progression) | — |
| ORF27* | 33717<—34076 (119) | 13.28 | 43.9 (0.003)  39.3 (0.082)  38.5 (0.14) | 33 (33/99)  30 (31/103)  27 (35/127) | CAL54007  XP_755283  AAM49603 | Ostreococcus tauri (Myosin class II heavy chain)  Aspergillus fumigatus Af293 (eukaryotic translation initiation factor subunit eIF-4F)  Staphylococcus aureus bacteriophage phi 3A (Phage tail fiber protein-like) | DUF1426 (102-111, 0.031, This family consists of several Banana bunchy top virus proteins of around 120 residues in length. is annotated a movement protein.) | — |
| ORF28 | 34128<—34940 (270) | 30.86 | 85.5(3e-150)  3e-16 | 37(60/161)  45(53/117) | ZP_00372803  GOS_5345935 | Wolbachia endosymbiont of Drosophila simulans (thymidylate kinase) | PF02223, Thymidylate_kin family (5-170, 1.7e-07), P loop containing nucleoside triphosphate hydrolases (1-188, 2.17e-18) | thymidylate kinase (dTMP Kinase) |

Footnote: The most significant homologues within the GOS data set and GenBank nr are listed for each ORF. ORFs marked with an asterisk have weak (E-value > 10^-3^) homology to virus structural proteins.

^a^ Data from the GenBank database (http://www.ncbi.nlm.nih.gov/).

^b^ Data from the Prosite database (http://cn.expasy.org/tools/scanprosite/).

^c^ Data from the Pfam protein family database (http://pfam.sanger.ac.uk/search).

^d^ Data from the Superfamily database http://supfam.mrc-lmb.cam.ac.uk/SUPERFAMILY/hmm.html).
