# Supplementary figures and images for: A Bacteriophage-Related Chimeric Marine Virus Infecting Abalone
Source: PLoS One. 2010 Nov 5;5(11):e13850. doi: 10.1371/journal.pone.0013850 (PMC2974647; doi:10.1371/journal.pone.0013850)

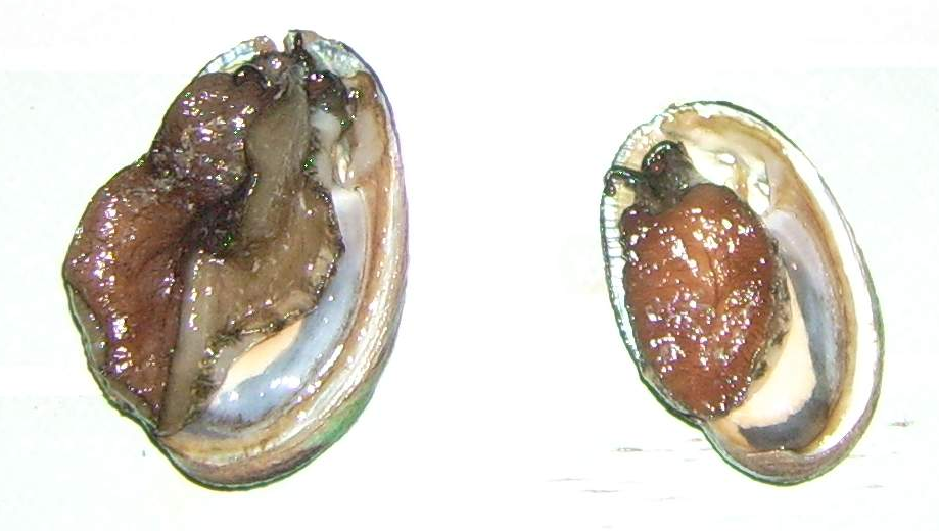

Supplement: Figure S1 — Typical clinical sign of sick abalone. Infected abalone H. diversicolor aquatilis with shrunken mantle and pleopod. (0.57 MB TIF) [file pone.0013850.s005.tif]

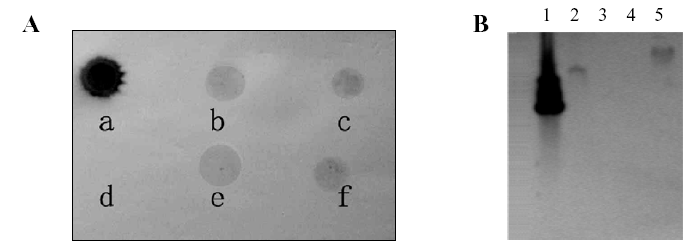

Supplement: Figure S2 — Dot/Southern blot hybridization confirmed that AbSV genomic DNA consisted in total DNA from infected abalone. (A) Dot blotting analysis. (B) Southern blotting analysis. Viral DNA and total DNA from infected abalone specifically hybridized to the DIG-labeled probe. No hybridization was detected to DNA from healthy abalone. Positive plasmid control (a and 1), total DNA from artificially infected abalones (b and 5), genomic DNA from healthy abalones (d, 3 and 4), isolated virus from infected abalone (e and 2), and total DNA from naturally infected abalone (f). (0.05 MB TIF) [file pone.0013850.s006.tif]

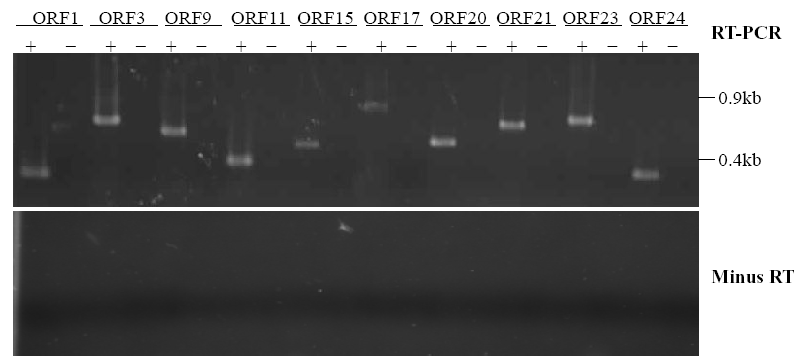

Supplement: Figure S3 — Electron microscopy of pathological ultrastructures of AbSV-infected abalone tissues (pleopod and alimentary canal). The myocyte of pleopod displayed diffuse alveolar damage (black arrows) in vertical section (A) and cross section (B) of myofilaments. c and d, In the hepatopancreas cells, the mitochondria cristae appeared to be fragmented (C) and dissolved accompanying marginalization of chromatin and extranuclear aggregates which might originate from fractured pieces (white double arrows) of ERs or other organelles (D). Bars represent 1Î¼m in panel A, 200nm in panel B and C, and 500nm in panel D. (0.06 MB TIF) [file pone.0013850.s007.tif]

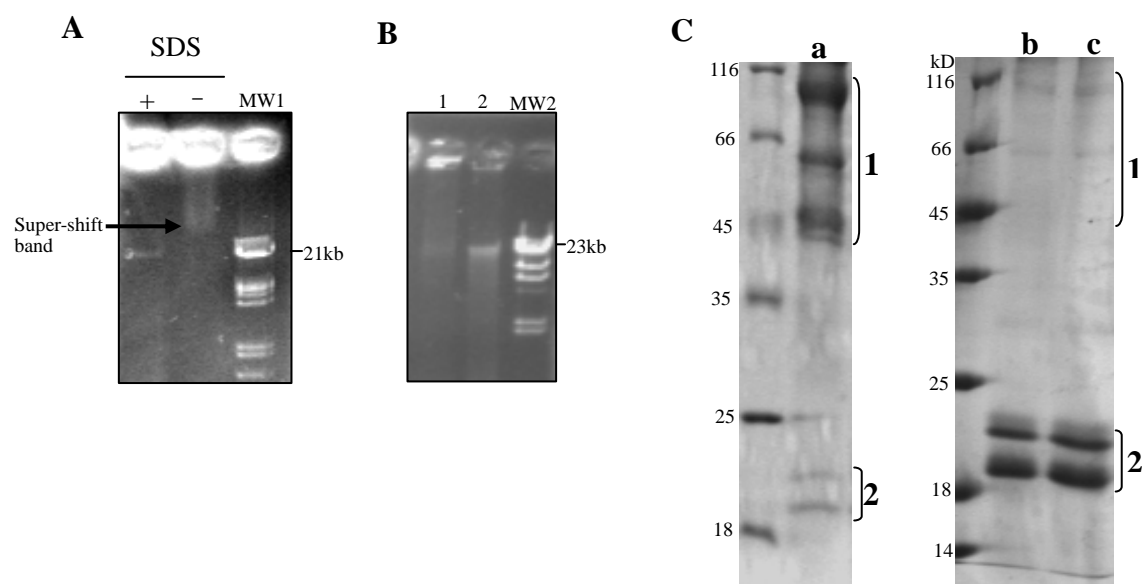

**D**

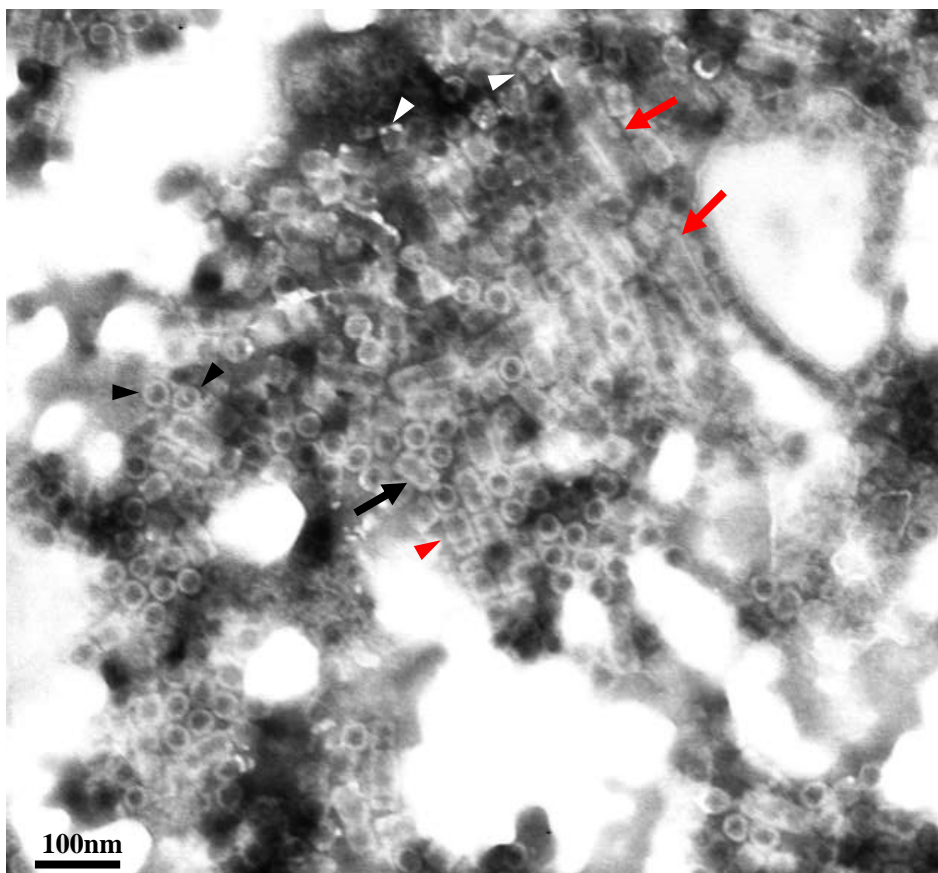

Supplement: Figure S4 — The AbSV genome is encapsided by hemocyanin-like particles. (A) The viral DNA was released from pellet suspension after SDS denaturation. (B) The extracted viral DNA from pellet obtained after PEG-mediated ultracentrifugation (lane 2) was more than one from pellet obtained through sucrose cushion ultracentrifugation (lane 1). The loading volumes were uniform. (C) Each sample lane was divided into two sections (brackets) and analyzed by mass spectrometry. Hemocyanin-like proteins (45 to 100kD region, section 1) act as a major component in pellet obtained after PEG-mediated ultracentrifugation (a); in pellet obtained through sucrose cushion ultracentrifugation (b and c), ferritin subunits (20 to 23 kD region, section 2) have the advantage of amount. (D) Electron microscopy of negatively stained purified particles from PEG-mediated ultracentrifugation. Aggregate distribution of hemocyanin-like particles was present under electron microscopy. Comparatively, the hemocyanin-like particles pellet sedimented by sucrose cushion ultracentrifugation sparsely distributed under electron microscopy (not shown). Note long rectangles (red arrows) like multidecamers, short rectangles (black arrows) which look like tetradecamers, circles (black arrowheads) or cubes (white arrowheads) resembling the typical top-views of or side-views of hemocyanin didecamers, respectively. (0.38 MB PDF) [file pone.0013850.s008.pdf]

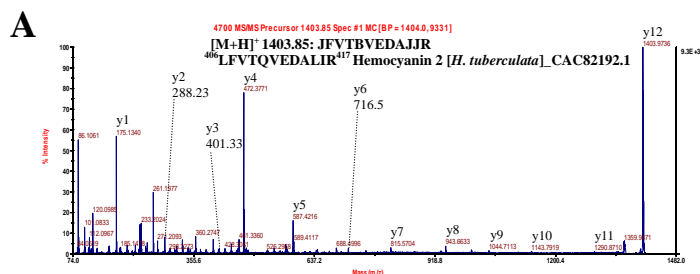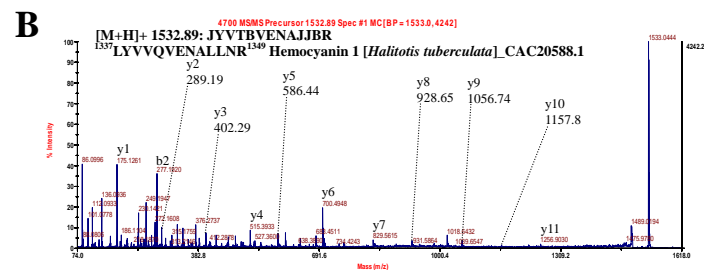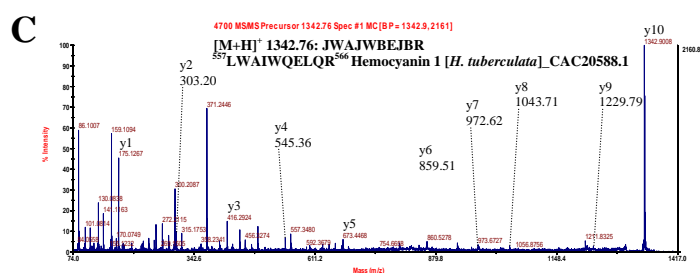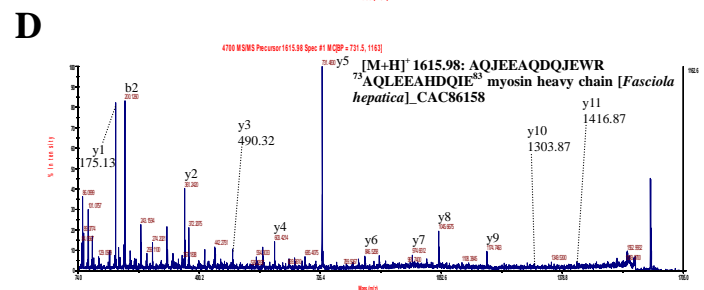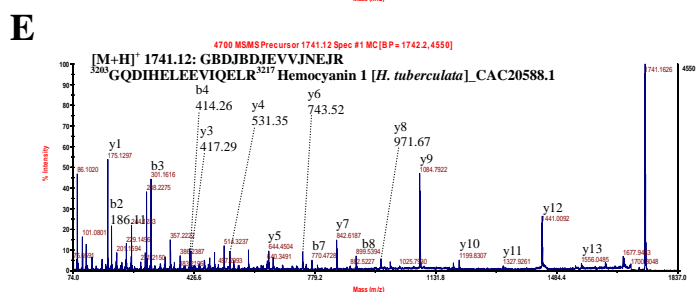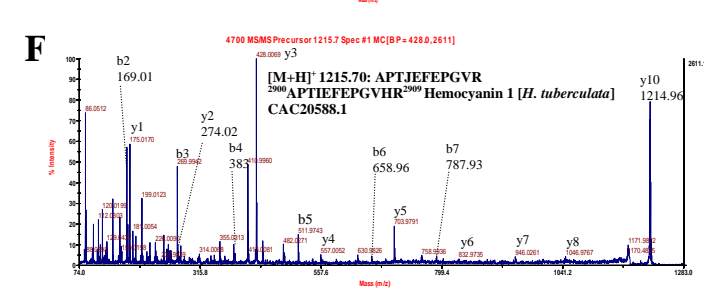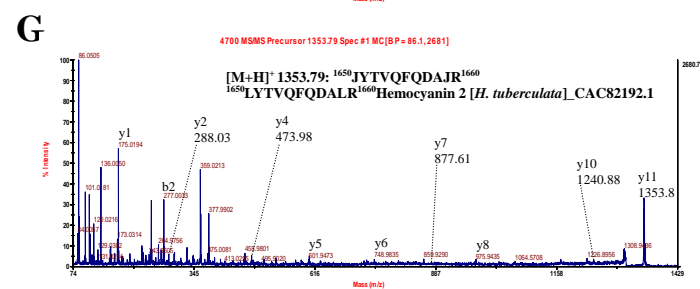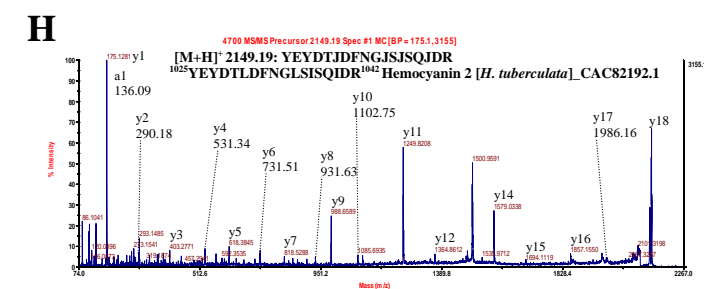

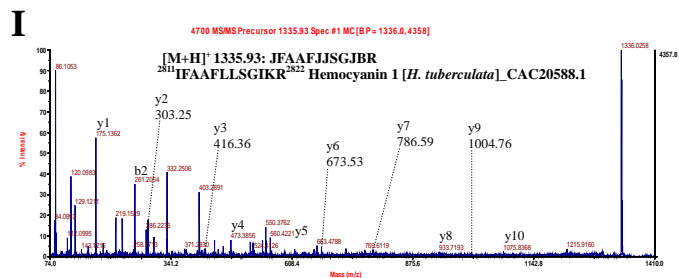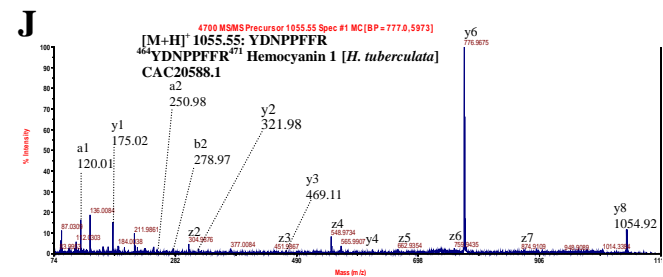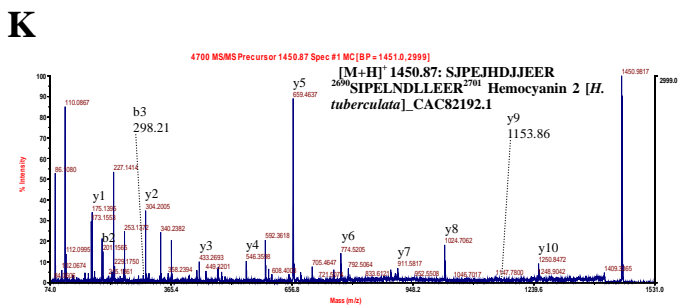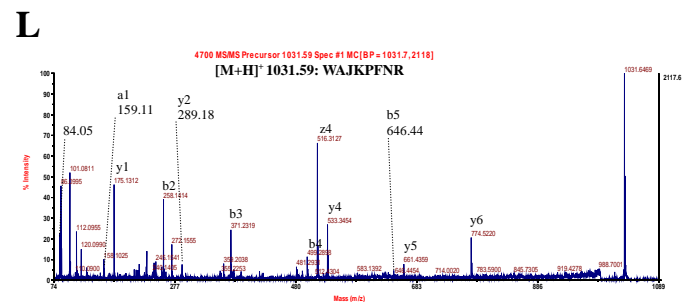

Supplement: Figure S5 — The representative MALDI-TOF/TOF MS spectra of trypsin-digested extract of modified hemocyanin subunits. These spectra were shown that identified peptides with [M+H]+ 1055.55, 1342.76, 1335.93, 1532.89 and 1741.12 corresponding to abalone hemocyanin type 1 subunit, and [M+H]+ 1353.79, 1403.85, 1450.87 and 2149.19 to abalone hemocyanin type 2 subunit. D, The peptide (m/z 1615.98) corresponded to myosin. F, The unambiguously identified sequence of peptide with m/z 1215.70 lacked one residue H comparing with corresponding region of abalone hemocyanin type 1 subunit. L, Due to the detectable iminium ions (m/z 84.05 and 101.08) in fragmentation spectrum, it indicated that this peptide contained lysine residue(s). The inferred sequence (WAI/LKPFNR) did not correspond to abalone hemocyanin subunits. Residue J can be either I (Ile) or L (Leu) and residues B stands for either Q (Gln) or K (Lys). (0.23 MB PDF) [file pone.0013850.s009.pdf]

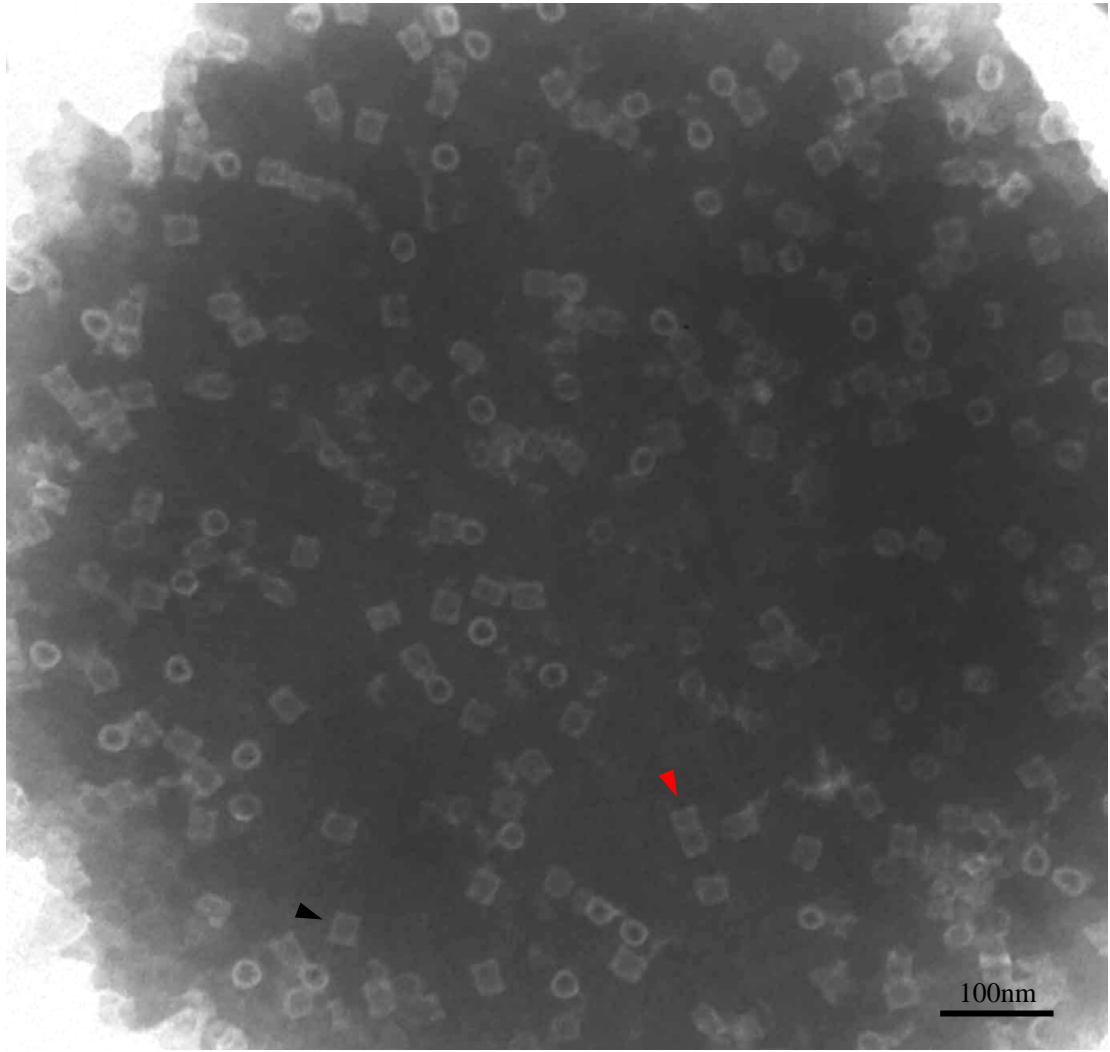

Supplement: Figure S6 — Electron micrograph of native hemocyanin from healty abalone. Not only didecamers (black arrowhead), but also tridecamers (red arrowheads) are observed. (0.25 MB PDF) [file pone.0013850.s010.pdf]

### A. Single-strand DNA binding protein

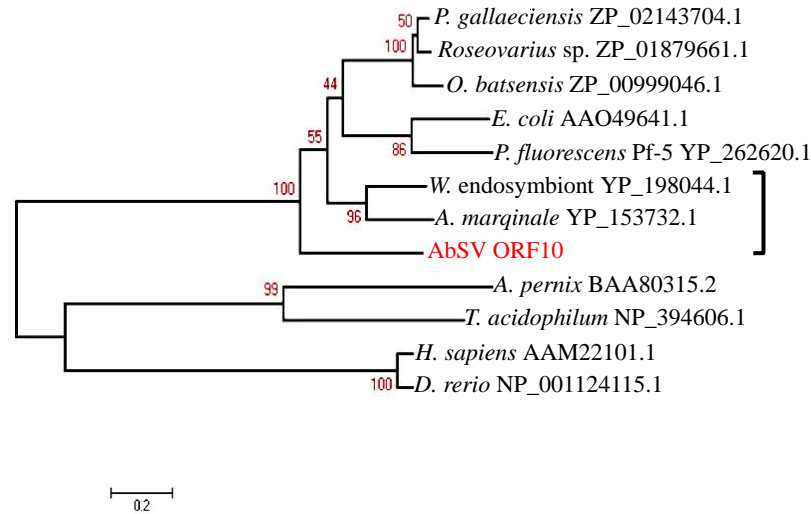

### B. Thymidylate kinase

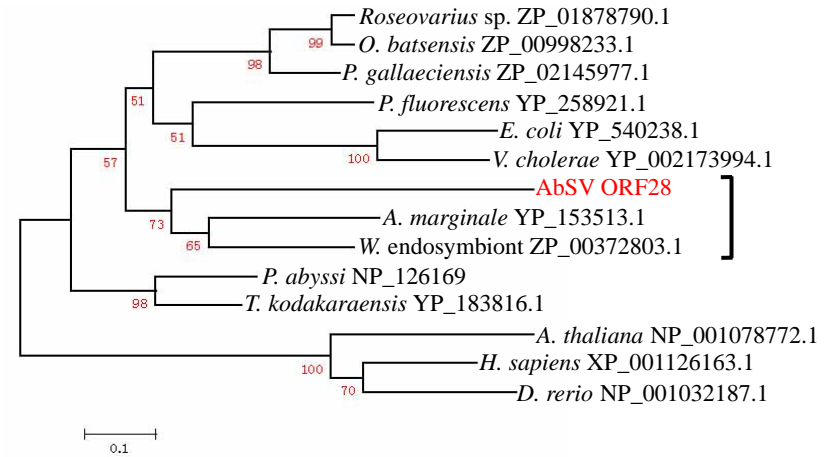

### C. Helicase

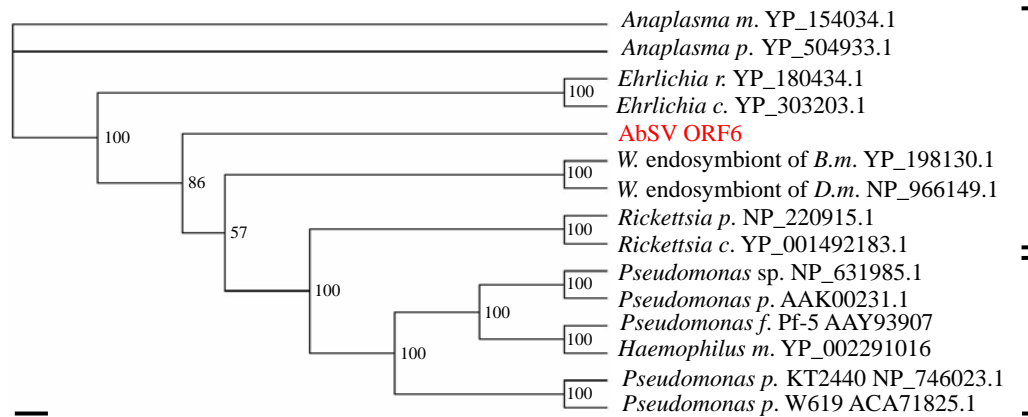

D. Primase

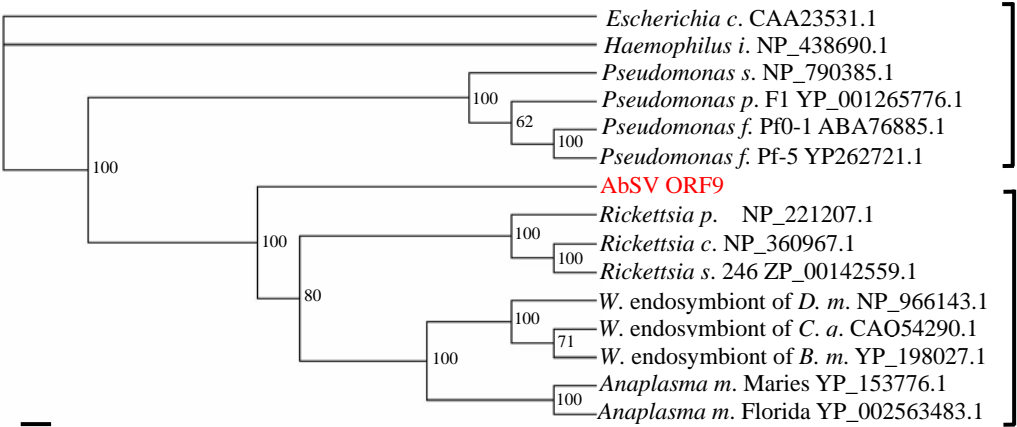

Supplement: Figure S10 — The phylogenetic trees of SSB protein, thymidylate kinase, helicase and primase. ORFs from AbSV are in red. The trees were inferred with neighbor-joining method based on SSB protein and thymidylate kinase (A and B, repectively); sequence data are the same as in Fig. 6b and 6a individually. And trees were constructed with the use of Mrbayes mixed model based on helicase and primase (C and D, respectively). These four genes correspond to origin of endosymbionts. Neighbor joining bootstrap value and posterior probabilities are indicated. Scale bars (C and D), 0.2 amino acid substitution per site. (0.07 MB PDF) [file pone.0013850.s014.pdf]
