# Supplementary material for: A Bacteriophage-Related Chimeric Marine Virus Infecting Abalone
Source: PLoS One. 2010 Nov 5;5(11):e13850. doi: 10.1371/journal.pone.0013850 (PMC2974647; doi:10.1371/journal.pone.0013850)
Supplement: Figure S7 — Identification of ferritin by MS. The proteins in viral suspension were denatured and separated on isoelectric focusing (4-7) IPG strips and then separated in the second dimension on a 12.5% SDS-PAGE (A). (B) A representative Q-TOF MS spectrum is shown that identifies spots 1, 2 proteins as the same subunit of abalone ferritin. (C) A representative MALDI-TOF/TOF MS spectrum, which identifies spots 3, 4 proteins as another subunit of ferritin. (D) The AbSV suspension was also separated on non-denaturing gels that was then was subjected to Prussian Blue staining for iron. It suggested that the amounts of iron were incorporated into the viral suspension. (0.23 MB PDF) [file pone.0013850.s011.pdf]

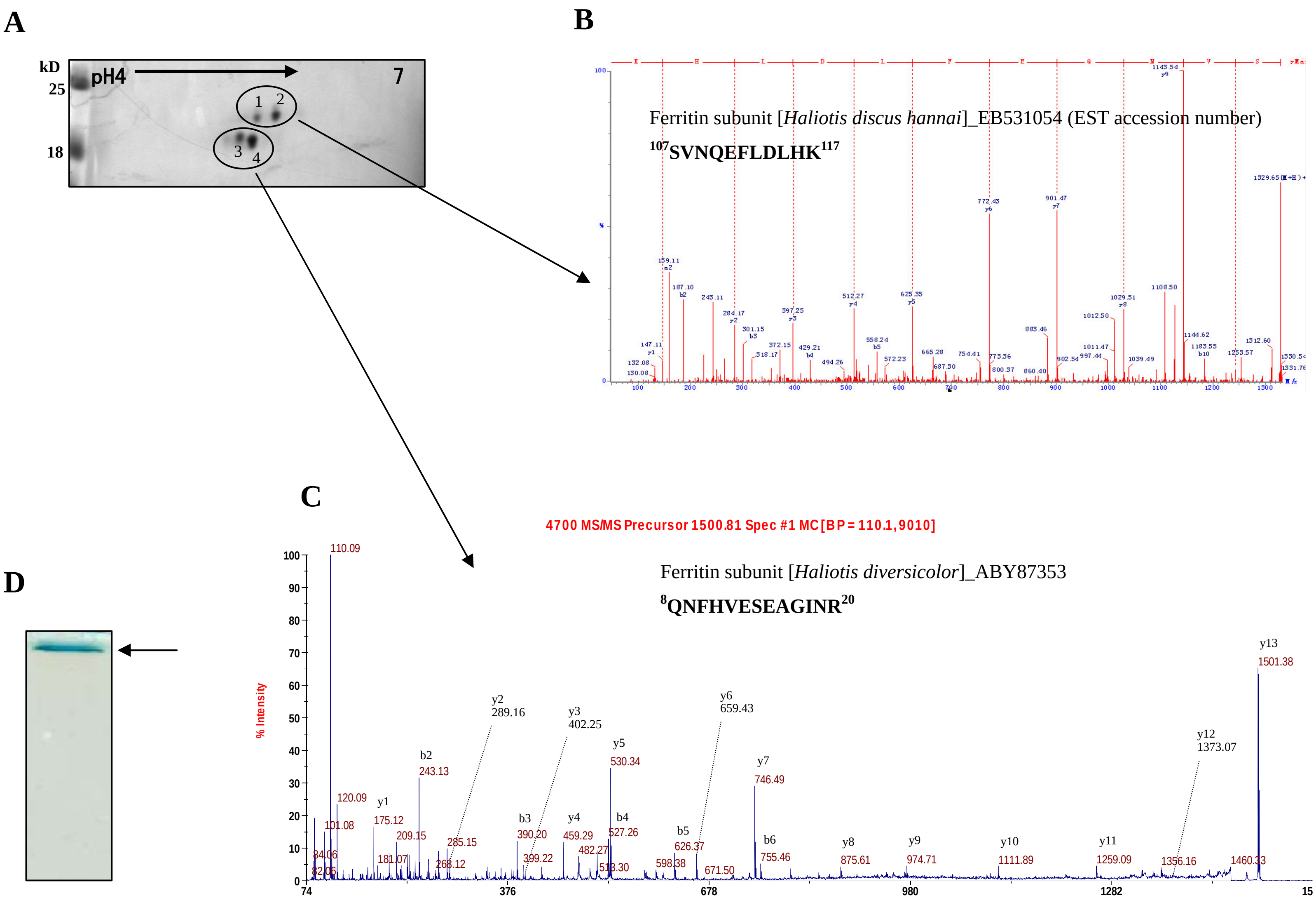
A
kD
pH4
7
25
18
1
2
3
4
B
Ferritin subunit [Haliotis discus hannai]_EB531054 (EST accession number)
107SVNQEFLDLHK117
C
4700 MS/MS Precursor 1500.81 Spec #1 MC[BP = 110.1, 9010]
Ferritin subunit [Haliotis diversicolor]_ABY87353
8QNFHVESEAGINR20
% Intensity
110.09
120.09
101.08
84.06
82.06
y1
175.12
181.07
209.15
b2
243.13
y2
289.16
285.15
268.12
b3
390.20
399.22
y3
402.25
y4
459.29
482.27
y5
530.34
b4
527.26
513.30
598.38
b5
626.37
y6
659.43
671.50
y7
746.49
b6
755.46
y8
875.61
y9
974.71
y10
1111.89
y11
1259.09
y12
1373.07
1356.16
1460.33
y13
1501.38
D
