# Supplementary material for: A Bacteriophage-Related Chimeric Marine Virus Infecting Abalone
Source: PLoS One. 2010 Nov 5;5(11):e13850. doi: 10.1371/journal.pone.0013850 (PMC2974647; doi:10.1371/journal.pone.0013850)
Supplement: Figure S8 — Identification of amino acid sequence modification of ferritin subunit from AbSV-infected H. diversicolor aquatilis. A, The peptide (m/z 1223.8) sequence was be definitely deduced from the typical Q-TOF-MS spectrum. B, The determined sequence has ambiguous homology with corresponding sequence of ferritin subunits of the listed marine species. But the amino acid residues in the corresponding positions of ferrtins within the listed marine species are identical. (0.17 MB PDF) [file pone.0013850.s012.pdf]

A

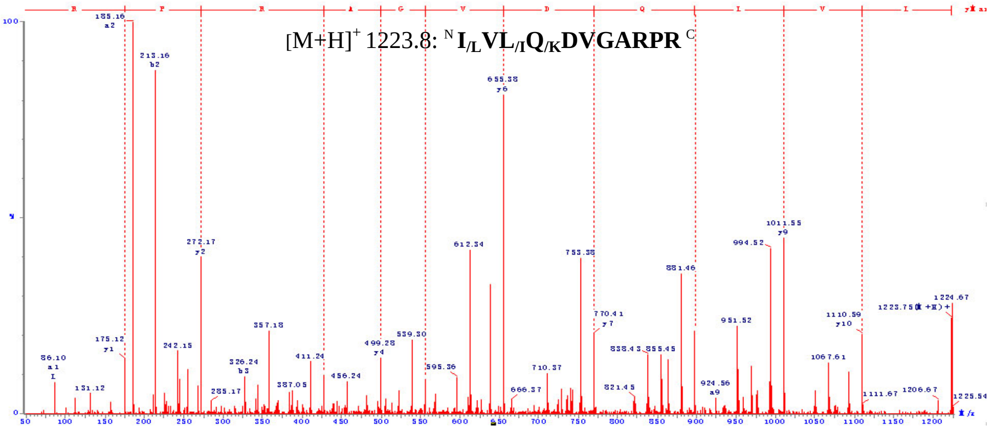

B

[M+H]+ 1223.8	IVLQDVGARP
*Haliotis diversicolor*_ABY87353.1	78 IVLQDI-KKP 86
*Haliotis discus hannai*_ABH10672.1	78 IVLQDI-KKP 86
*Haliotis dicus discus*_ABG88846.1	78 IVLQDI-KKP 86
*Pinctada fucata*_AAQ12076.1	78 IVLQDI-KKP 86
*Asterias forbesii*_AAB60883.1	76 IVLQDI-KKP 84
*Nematostella vectensis*_XP001624357.1	76 IVLQDI-KKP 84
*Pseudorca crassidens*_BAG82923.1	78 IVLQDI-KKP 86
*Salmo salar*_ACI68639.1	79 IVLQDI-KKP 87
