# Supplementary material for: A Bacteriophage-Related Chimeric Marine Virus Infecting Abalone
Source: PLoS One. 2010 Nov 5;5(11):e13850. doi: 10.1371/journal.pone.0013850 (PMC2974647; doi:10.1371/journal.pone.0013850)
Supplement: Figure S9 — Alignment of putative thymidylate kinase (N-terminal) domain, single-strand DNA binding protein (N-terminal) domain, helicase domain (P-loop region) and primase (N-terminal) domain among AbSV and other organisms. The regions with gray background in panel B represent ssDNA binding sites. The identical residues were denoted by asterisks, and conserved or semiconserved residues were denoted by double dots or single dots. (0.06 MB PDF) [file pone.0013850.s013.pdf]

A. Thymidylate kinase

Nucleotide phosphate-binding motif (Walker A)

```
A.marginale        MFITFEGIDGCGKTTQAVLLAKYLSDLYGEHRVVLTREPGGTSLNELIRGALLGLTDY------KLDGITELMLFIAMRRESFVKVVLPGLLAGKIVISDRFTDS
W.endosymbiont     MFITFEGIDGSGKTTQSKLLANHFKQIHGENNVVLTREPGGTNFAEKIRGVLL--TN-------NIDPISELLLLISMRREHMKKLILPALAEGKIVICDRFIDS
AbSV               MFITIEGIDKIGKSTVAQGLQRKFSQ-YSDNSIVLTHEP--FNFNGLSN--FL--KKY------QLSSKVEILIQTAARIEHATRLINPAISSGSIVICDRFIDS
O.batsensis        LFLSFEGIDGSGKSTQARMLAEALRA--EGRDVVLTREPGGSPGAEEIRALVLQGDPD------RWSAATEILLFTAARRDHLERTIRPALDRGAVVICDRFADS
roseovarius        LFLSFEGIDGSGKSTQARRLAETLRA--KGRDVVLTREPGGSPGAEEIRRLVLEGDKD------RWSAETEILLFTAARRDHLERVIAPALARGAVVICDRFADS
P.gallaeciensis    LFVTFEGIDGSGKSTQCRLLAEALQA--QGIEVVLTREPGGSDGAEEIRRLVLEGDPD------RWSAETELLLFTAARRDHMERRILPALAAGKVVVCDRFADS
E.coli             KYIVIEGLEGAGKTTARNVVVETLEQ-LGIRDMVFTREPGGTQLAEKLRSLVLDIKSVGDE---VITDKAEVLMFYAARVQLVETVIKPALANGTWVIGDRHDLS
Vibrio             KFIVIEGLEGAGKSTAIQVVVETLQQ-NGIDHITRTREPGGTLLAEKLRALVKEEHPG--E---ELQDITELLLVYAARVQLVENVIKPALARGEWVVGDRHDMS
P.abyssi           YFVVLEGIDGSGKTTQAKLLAEWFEE--QGWDVLLTKEPTDTEFGRLIRELVL---KNSIIDGSRISYEAEALLFAADRAEHVKKVILPALEKGKVVICDRYLYS
T.kodakaraensis    MFIVLEGIDGAGKSTQAKLLAEWFEN--RGYEVVLTKEPTDTPFGKLIRRLVLTGGKEGIIDGAKISHEAEALLFAADRAEHVDKLIKPSLEAGKVVISDRYFYS
H.sapiens          ALIVLEGVDRAGKSTQSRKLVEALCA--AGHRAELLRFPERSTEIGKLLSSYLQKKSD-------VEDHSVHLLFSANRWEQVP-LIKEKLSQGVTLVVDRYAFS
D.rerio            ALIVLEGVDRAGKTTQCQKLVQAIQQ--SGRAAEIMRFPDRTTKIGQLISSYLEKKSN-------LEDHTVHLLFSANRWEMVP-VMKQKLEEGINLVVDRYAFS
A.thaliana         ALIVLEGLDRSGKSTQCAKLLSFLNG--LGHPTELWRFPDRETSVGQMISAYLSNKSQ-------LDDHTIHLLFSANRWEKRS-LMEEKLKTGTTLIVDRYSYS
                     : :**::  **:*    :   :             : *              .                   ::  : * :     :   :  *  :: **.  *
```

B. Single-strand DNA binding protein

```
A.pernix           EGDRTRIMDLREGLRNVS----ISGRVLETGEPKMVETKRG-PATLSEAVVGDESGRVKVTLWGSHAGTLKEGEAVRIEGAWTTSYRGKVQVNVGRESTIEKVDSEDVPQAEDIPEEMPE
T.acidophilum      MEDITKIKDLTPSSRRVN----VVGKVLSVGEPKTIQTKFGDQRSVTEVTIGDDTGKVILSLWGDQASQARAGETLAIGNGYVSLVRGHIRLNVGKYGSLSVSDEEVTEVNEDFDASEKE
W.endosymbiont     MSNGTVNKVILVGNLGRN----PEIRTMQNGK-EMANFSIATSEGWTDKFSGVRSEKTEWHNIVIFSEGLVKVVKDFVRKGSKVYIEGSLKTRKWTDQNGSER-YTTEVVLQNFNGTLTL
A.marginale        MSSLGVNKVILIGHLGKD----PDVRVMQNGK-EMASFSLATSESWLDKASGARTEKTEWHSIVVFSEGLVRVVKTCARKGSKVYIEGSLRTRKWSDQNGNDR-YTTEVVLQGFNSALCL
AbSV               MKN-SINKTILVGRTGKD----PVIRTMNNGS-KVANFPLATSESWIDKTKNERREKTEWHNIVIYGDGLASIAEKFVRKGSRLYIEGAIQTRKWVDVENNQR-TTMEIVLQGFNSTLVI
O.batsensis        MAG-SVNKVIIVGNLGRD----PEVRSFQNGG-KVCNLRIATSENWKDRNTGERRERTEWHSVAIFSEPLARIAEQYLRKGSKVYIEGQLETRKWQDQSGQDR-YSTEVVLRPYRSELTL
P.gallaeciensis    MAG-SVNKVILIGNLGRD----PEVRSFQNGG-KVCNLRIATSETWKDRNTGERREKTEWHSVAIFSEGLVRVAEQYLRKGSKVYIEGQLQTRKWQDQSGQDR-YSTEVVLQGIGGTLTM
Roseovarius        MAG-SVNKVILIGNLGRD----PEVRTFQNGG-KVCNLRIATSETWKDRNTGERRERTEWHSVAIFNEGLVRVAEQFLRKGSKVYIEGQLQTRKWQDQSGQDR-YSTEVVLQGFGSTLTM
E.coli             MAVRGINKVILVGRLGKD----PEVRYIPNGG-AVANLQVATSESWRDKQTGEIREQTEWHRVVLFGK-LAEVAGEYLRKGAQVYIEGQLRTRSWED-NGITR-YVTEILVK-TTGTMQM
P.fluorescens      -MARGVNKVILVGTCGQD----PEVRYLPNGN-AVTNLSLATSEQWTDKQTGQKVERTEWHRVSMFGK-VAEIAGEYLRKGSQVYIEGKLQTREWEK-DGIKR-YTTEIIVD-MQGTMQL
H.sapiens          MFAKGKGSAVPSDGQAREKLALYVYEYLLHVGAQKSAQTFLSEIRWEKNITLGEPPGFLHSWWCVFWDLYCAAPERRDTCEHSSEAKAFHDYSAAAAPSPVLGNIPPNDGMPGGPIPPGF
D.rerio            MYAKGKGAVVPSDSQAREKLALYVYEYLLHVGAQKSAQTFLSEIRWEKNITLGEPPGFLHSWWCVFWDLYCAAPDRRETCEHSSEAKAFHDYSAAAAPSPVMGNMPPNDGMPGGPMPPGF
                          :  .    .       . :               .                                             ..
```

C. Helicase

P-loop motif (Walker A)

```
Rickettsia p.                 181  ASINRAMKNNDHVIGISTGLLDLDNKLFGFHNSDLIILAGRPSMGKTAFAINLALNTCNNMRLKNIRDNQEIKSVGFFSLEMSSEQLTTRLLSLCAEIDSTALRTGMLSEDKYNRLRKEANTLSELQFFIDDTPALSISAIRTRARRMKRKHNLGILFIDYLQLIRGVSK--SENRVNEISEITQGLKAIAKELNIPVIA 379
Rickettsia c.                 189  ANINRAMKNNDHVIGISTGLIDLDNKLFGFHNSDLIILAGRPSMGKTAFAINLALNACNNMRLKNIRDNQEIQSVGFFSLEMSSEQLTTRLLSMCAEIDSTSLRTGVLGEEKYNRLRKEANTLSELQFFIDDTPALSITTIRTRARRMKRQHNLGILFIDYLQLIRGVSK--AENRVSEIAEITQGLKAIAKELNIPVIA 387
W.  endosymbiont  of  B.m.    183  KKISTLKNNPEALG-ITTGLQDLNQLLGGLQKSDLLILAARPSMGKTALALNIALNACKVLQKR----ADEQHYVAFFSLEMSAEQLTTRLITIDSGISYYKALTGRISDFELHEFINASAELSELPFIIDDTPALSISALRTRIRLLYQLYNVEAVFIDYLQLIRGTTKRSNENRVQEISEVTQGLKAIAKELNIPVVA 378
W.  endosymbiont  of  D.m.    183  EKISTLKNNPEALG-VTTGLQDLNQLLGGLQKSDLLILAARPSMGKTALALNIALNACKMLQKR----ADKQHYVAFFSLEMSAEQLTARLITIDSGISYYKALTGRISDFELHEFINTSTELSELPFIIDDTPALSISALRTRIRLLYQLYNVEVVFIDYLQLIRGTTKRSNENRVQEISEVTQGLKAIAKELNIPIVA 378
Anaplasma m.                  184  KKIEAAKGGANALQGVSVGLRDLDQLLNGLQNSDLIVVAARPSMGKTALALNMALSACKSLKN-------DGKSVGFFSLEMSAEHIASRIISIESEISSYKALSGKISSTDLNQVLKASEKICDLPLIVDDSSVLSISGLRTRIRRMHQLHNVGVVFVDYLQLIKGTTKRSGENRVQEVSEVTQGLKAIAKELNVPLVA 377
Anaplasma p.                  183  KKIESARHNKDALQGITVGLKDLDLLLNGLQNSDLIILAARPSMGKTALALNMALSACKSLKE-------KGQSVGFFSLEMSAEHIASRLISIESEISSYKALSGKISSGDLQQVLRASENICDLPLVIDDSSALSISGLRTRIRRMHQLHNVGVIFIDYLQLIKGTTKRSGDNRVQEVSEVTQGLKAIAKELNVPLVA 376
Ehrlichia r.                  179  KRIEFAKSNEGTLQGISVGLRDLDQLLNGLQKSDLIILAARPSMGKTALALNMALNACKLLHSK---DQYNGMHIGFFSLEMSAEQLASRVIAIESEVSSYKALSGKISGEELNKVIDASTKVCNLPLIIDDTSSLSISSLRTRIRRMHQLYNLGAVFIDYLQLVKGTTKRSGENRVQEVSEVTQGLKAIAKELNIPVVA 376
Ehrlichia r.                  181  KKIEFAKNNEGVLQGVSVGFRDLDQLLNGLQKSDLLILAARPSMGKTALALNMALNACKILSNS---SQYNGQHIGFFSLEMSAEQLASRVIAIESDVSSYKALSGKISGEELKKVIDASTKVCSLPLIIDDSSSISISSLRTRIRRMHQLYNLEAVFIDYLQLVKGTTKRSGENRVQEVSEVTQGLKSIAKELNIPVVA 378
AbSV                          168  EGLDEIREALNENHCISTKLHNLDRTINGLRLGKLVTLGARTSMGKTSLALKIMNNLSKELHIKN-CKKNSTTSVAYLSYEMHSEELQGRVLSFESKVPVHRIFSPDCTTCDMIEIENAQKVLCELPQHIKFLEDRTLDHLVAEIEDLQEKHNCKYFFVDHIHLLS-LTGDISNYRVHEMSVISGRLKTLTLKNNITIVC 366
                                     :.           ::.  :  :*:   : *:: ..*: :..*.*****::*:::  .  .: :        .    :.::* ** :*.:   *:::: : :       :       .   ..        :..*    :.      ::   : :.  . : .  :*    .*:*:::*:    :     : ** *:: ::   **::: : *:.::.
```

D. Primase

```
                                            Zn-finger                                                                                                                                    Catalytic core
Anaplasma m. Maries          KIKLIKRGSNHYVGLCPFHSEKTPSFHVNCSNDMFYCFGCGVHGDVVQFVSDIDGLSFREAIEYLAQVYGVSLPAKAGRG-----EADFLYELMDYAARWFVEQLSKSPTALSYLRSRGIDEKTIRKFRLGYVPVSG-IKTCFASSQISFEKVRDAGLLTK----NFQDCLYNRLVFPICSATGRVIAFGGR-SVSDKHSPKYLNSAENALFKKRESL 239
Anaplasma m. Florida         KIKLIKRGSNHYVGLCPFHSEKTPSFHVNCSNDMFYCFGCGVHGDVVQFVSDIDGLSFREAIEYLAQVYGVSLPAKAGRG-----EADFLYELMDYAARWFVEQLSKSPTALSYLRSRGIDEKTIRKFRLGYVPVSG-IKTCFASSQISFEKVRDAGLLTK----NFQDCLYNRLVFPICSATGRVIAFGGR-SVSDKHSPKYLNSAENALFKKRESL 239
W. endosymbiont of D.m.      KVRLIKRG-DSFVGLCPFHNEKTPSFSVSNTKGLYYCFGCLASGDAFEFISQTEGLSFKEALEKLASVTGVELPKNLSITK----EDNKLFLALDLAANWFAQ---KNQGVVDYLKQRKILPKIIDKFKIGYAPSSG-LKEYLNSSGIEDKILIDIGLVNK----NFHDYFYDRLIFPIQSIAGRVIGFGGR-ALSSEQQPKYLNSPESQLFKKRENL 223
W. endosymbiont of C.q.      KVRLIKRG-DSFVGLCPFHNEKTPSFSVSNVKGLYYCFGCSAHGDAFEFISQTEGLSFKEALENLASVAGVELPKNLSFAK----ENDKLFSALNLAASWFAQ---KNRGVMAYLRQRKISLKIIDKFRIGYAPSSG-LKEYLNSSGIKDEILIDVGLINK----NSRDYFYDRLIFPIHNITGKVIGFGGR-ALNSEQQPKYLNSPESQLFKKRENL 223
W. endosymbiont of B.m.      KVRLTKRG-DSFVGFCPFHSEKTPSFLVSNTKGSYHCFGCSAHGDVFNFISQTEGLSFKEALERLASVVGVELPKTLNLAK----ENGELFSTLNLAASWFVQ---KNQGIIAYLKQRKISPKIIDKFKVGYAPSSG-LKEYLNSLGIEDKILIDVGLINK----NSRDYFCNRLIFPIYNIAGKVIGFGGR-ALSSKQQPKYLNSPESQLFKKRENL 223
Rickettsia c.                KVALTRKS-GNYVGLCPFHQEKTPSFTVSNSKRFFYCFGCKAAGDVIKFTSNISGLSYNESAIKLAIDYGIEIPKLTAKQKEFYEESDEILNILELANKFFRT--QLTPEILHYLHERGITEETVKEFSIGFAPKNNKFEKFFHDKNIDIIKLGKAGLIGKRENGEIYNLFSNRITIPIRNIYNKIVGFGGR-VLG-EGLPKYLNSPETTVFQKSETL 236
Rickettsia s.                KVALTRKS-GNYVGLCPFHQEKTPSFTVSNSKRFFYCFGCKAAGDVIKFTSNISGLSYNESAIKLAIDYGIEIPKLTAKQKEFYEESDEILNILELANKFFRT--QLTPEILHYLHERGITEETVKEFSIGFAPKNNKFEKFFHDKNIDIIKLGKAGLIGKRENGEIYNLFSNRITIPIRNIYNKIVGFGGRVLG--EGLPKYLNSPETTVFQKSETL 236
Rickettsia p.                KLALTRKS-SNYVGLCPFHQEKTPSFTVSDSKRFFYCFGCKASGDVIKFTSNISGLSYNESAIKLANDYGIEIPKLTVKQKEFYEESDNILNILELANKFFRT--QLTPEILNYLYKRNITETTIKEFSIGFAPRNNKFEKFFLDKKIDITKLGQAGLIGKCKNGKIYNLFSNRITIPIRNIYNKIVGFGGRVLG--NELPKYLNSFETIVFQKSDIL 236
AbSV                         HVRLTERS-GRLFGLCPFHEEKTPSFIVSDELGKFYCFGCNAHGDVFDFLSKLLGISDHEALEYLAKDTGTIIDSKPDTKT----------MFFAKVAEIFMVSLLDNVEALNYLKKRGVDQTAIKLWSIGFFSLEK-LKEFTDNLTSEARESFSDFVQLT-----ALNQLANRIIFPIHDKTGAIIAFSGRNLDIETQTPKYINTKNTESFKKGSCF 220
                             :: * .:. .  .*:****.****** *.     ::**** . **...* *.  *:* .*:   **   *  :                    :  .   *      .   : **:.*: :    :  : :*: . .  ::    .    .     .  : .       : : :*: :** .  . ::.*.**        ***:*: :.  *:* . :
```
