# Supplementary material for: A Bacteriophage-Related Chimeric Marine Virus Infecting Abalone
Source: PLoS One. 2010 Nov 5;5(11):e13850. doi: 10.1371/journal.pone.0013850 (PMC2974647; doi:10.1371/journal.pone.0013850)

A. Alignment of resovase (serine recombinase)

```
Pseudomonas sp.              -MLIGYARVSTDDQLLDLQRDALEKAGCERVFEDT-ASGAKAERVGLTALLTTLRRGDTVVIWRLDRLGRSLKDLIRLVEQLDATGVGLRSLQESI---DTASSGGRLIFHLFGALAEFERNLIRERTQAGLSAARARGRK-GGRKKRLDPAK--QELALSLYHERKHTVAEICRLMGIGRSTLYNYL 180
Pseudomonas s.               -MLIGYARVSTDDQLLDLQRDALEKAGCERVFEDT-ASGAKAERIGLTALLATLRRGDTVVIWRLDRLGRSLKDLIRLVEQLDAAGVGLRSLQESI---DTASIGGRLVFHLFGALAEFERNLIRERTQAGLSAARARGRK-GGRKKRLDPAK--QELAMRLYHERKHTVAEICRLMGIGRSTLYNYL 180
Nitrosospira m.              -MKIGYARVSTDDQNLDLQLDALTKAGCEQVFTDHGVSGATIEREGLSQAIAAVGKGDVLVVWKLDRLGRSLSFLIELIEKLRNEGAGFESLSDGI---DTTTAGGKLVFHIMGALAEFERSLISERSKAGMQAARRRGKH-IGRPHKLSREQ--ISHAAQMIQEGRETVSGMAGLLSVDRATLYRAL 181
Staphylococcus h.            -MKIGYARVSTGLQNLNLQEDQLNQYGCEKIFSDH-ISGSKSKRPGLDKAIEFARAGDTIVVWRLDRLGRNMEDLITLVNELNNRGVSFHSLEENITM-DKSSSTGQLLFHLFAAFAEFERNLILERSSAGRIAARARGRY-GGRPEKLNKQD--LKLLKTLYDNG-TPIKTIAEQWKVSRTTIYRYL 181
Staphylococcus a.            -MKIGYARVSTGLQNLNLQEDRLNTYGCEKIFNDH-MSGSKSKRPGLDKAIEFARSGDTIVVWRLDRLGRNMEDLITLVNELNERGVSFHSLEENITM-DKSSSTGQLLFHLFAAFAEFERNLILERSSAGRIAARARXRY-GGRPEKLNQKD--LNLLKTLYDNG-TPIKTIAEQWHVSRTTIYRYL 181
Methanococcus m.             -MIFGYARVSTQDQNVDRQLDELKAAGCQKIFYEK-ISGTKMERPEFLRMLDQVRSGDLIIITELTRLSRSTKDLLNVVEMLEEKGVSIKSLKESW-L-DTSSAHGKLLFTFFAGISQFERDIISERVKSGLQAARARGRL-GGR-KPADPKT--IDIAVRMWKSKEYSITEILKTCGISRRTLYKYL 180
Bacillus c.                  -MIYGYARVSAQDQNLDTQIEQLLKYGVDKIVKEK-ISGVSQGKIELDDLLSRLIKGDTLVVTRMDRLGRNTIQLLQFVEHLREKGVHFAVLNLGI---DTRTPTGKFFLTVMSAFSELDREMIKEKQIAGIKLAKQKGVY-RGRIKKYTEQHAGMNHAIELRQQTKKTIKEICAITGVSQAALYRKL 182
Bacillus a.                  -MIYGYARVSAQDQNLDTQIEQLLKYGVDKIVKEK-ISGVSQEKIELDDLLSQLLKGDTLVVTRMDRLGRNTIQLLQFVEHLREKGVHFAVLNLGI---DTRTPTGKFFLTVMSAFSELDREMIKEKQIAGIKLAKQKGVY-RGRIKKYTEQHAGMNHAIELRQQTKKTIKEICAITDVSQAALYRKL 182
AbSV                         -VLYGYARVSKQKQNLTTQIRLLNKAGCKQIFYEK-ISGAKDKKVELGKLISVLQKGDVVVVAHLDRLGRNLVTTLRTIEQINNKGASIKALDFNL---DSGTAMGDLMIKFMAFFAELERYFAKMRSEERLANLTKKQRSSLGRKAVLDKKT--IKKYYQQYLDRKTTVAAITEKLGITRASFYNAL 181
Haemophilus i.               MALIGFARVSTQQQDLSEQIQVLKEYGCKKIFSGKHSGKAEKNKAQLDELLDYIREGDVVAVTKLDRLGRSLAQCLNTLELFKNKNIGFVAIQQGIDTRTQNNPMGMALIQLLGIFAELERSFIVERTQEGKRTKLASGHK-NALGGRPPKVTDKIRKKIYADFKAGDSISTVMKRYDLSKSTVARLK 187
                                    *:****   * :  *   *   * .::.     .     :  :   :     ** : : .: **.*.    :  :: :  .   :  :. .      .  *  .: .:. :::::* :   :              .                  .   .:  :     : : :. .
```

B. Alignment of terminase large subunit

```
Pseudomonas putida                FDEASAIADLVWEVAEGALTDADTEIIWAAFGNPTRNSGRFRECFTKFKHRWRHRQ--VDSR-----TVDGTNKTQIAKWIADYG-EDSDFVRIRVRGMF 273
Enterobacteria phage epsilon 15   FDEASNIADLVWEVAEGALTDEDTEIIWVAFGNPTRNTGRFRECFRKYKHRWKCAQ--IDSR-----TVEGTNKQQLQKWVDDYG-EESDFVKVRVRGIF 272
Photobacterium p. SS9             FDEASAVPDKIWEVAEGGLTDG--EPFWFVFGNPTRNSGRFRECWRRFRQRWNRKQ--IDSS-----TVQVTNKKKISEWESDYG-EDSDFYRVRVKGVF 289
Ehrlichia r.                      VMQRLHSDDLAGYLLNS--SNGWHHLKIPAIAD-------CDYSFKLTANSYVKGE--VLEN-------YKEPADCLAKLEHEIG-SYNYHAQYLQEPIP 233
Rickettsia b.                     VMHRLHTEDLTGYLLSKKLHNKWHVLSLPAIAEKKHIIYSIAYPWKFKYKKKVKNI--LHIRKEGDFLYWKYGKKYINELKSELG-SYAFAAQYQQNPIN 279
Clostridium phage phiCD27         VDEASGVADPIMEAILGTLSGA--ENKLLLCGNPTRTSGTFYDSHNRDRDLYKTFK--VSSLD-----SPRTSKDNIEMLKRKYH-EGSDPWRVRVLGEF 236
Enterobacteria phage p1           IDEASGVSDKAFSVITGALTGK--DNRILLLSQPTRPSGYFYDSHHRLAIRPGNPDGLFTAIILNSEESPLVDAKFIRAKLAEYGGRDNPMYMIKVRGEF 258
AbSV                              FDESTYFNDHVWQALENMWTQG--QVLCFCTGNPSHDNNNYFARLFNKSLHKKDSLWLTRCVSLLELPLKYRNDARARYIEEHYG-KTHPRYIASVLGQF 282
                                  . .     *                  .:                                                       .

Pseudomonas putida                PRASDLQLIPTDWVAEAMRR--DGVYGLD-DALVCGIDIARGGMDNNVIR 320
Enterobacteria phage epsilon 15   PDASELQFIPTGLTDEAMKRVVTAAQVAH-APVIIGVDPAYSGVDDAVIY 321
Photobacterium p. SS9             PSASSNQKISGALLEAAMSR--TAHVIPG-SPRVMSLDVARGGGDNCVFR 336
Ehrlichia r.                      QGSSLLNMEDISFYENLPEKFD--YLVQS-WDTAIKISED----SDYSVC 276
Rickettsia b.                     LSSGIIKYIWFQRYNQTLYNHENTNITQS-WDTASSVNYN----SDYSIC 324
Clostridium phage phiCD27         PKGESDSLISLEAVETSTIR--EVNISND-YILNIGADIARYGDDETIIA 283
Enterobacteria phage p1           PKSQDGFLLGRDEVERATRRKVKIAKGWG-WVACVDVAGGTGRDKSVINI 307
AbSV                              PKKNTCNPFDITAISEAMEREVREEFIHHPVIMGIDVSISANNGSASAIC 332
                                                  .                       .
```

C. Bayesian tree of terminase large subunit

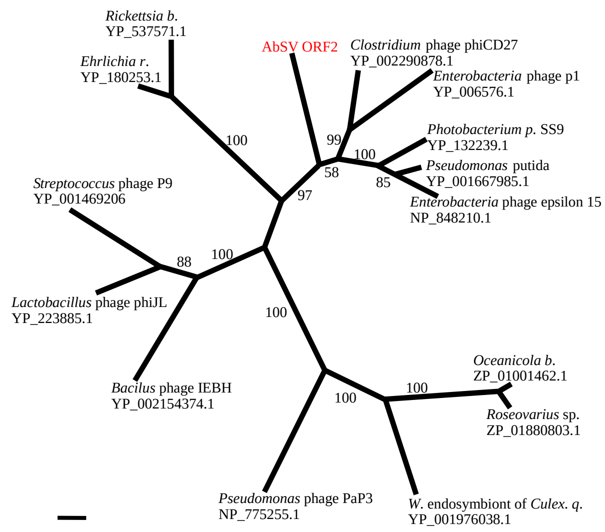

Supplement: Figure S11 — Alignments of resolvase functional domain and reserved domain of terminase large subunit, and Bayesian tree for terminase large subunit. (A) The regions with gray background corresponds to catalytic residues, yellow background to DNA binding sites. (B) and (C), AbSV is closer to the phage clade in consensus tree of terminase large subunit. Posterior probabilities are shown above each branch. Scale bar, 0.2 amino acid substitution per site. (0.05 MB PDF) [file pone.0013850.s015.pdf]
