# Supplementary material for: A Bacteriophage-Related Chimeric Marine Virus Infecting Abalone
Source: PLoS One. 2010 Nov 5;5(11):e13850. doi: 10.1371/journal.pone.0013850 (PMC2974647; doi:10.1371/journal.pone.0013850)
Supplement: Figure S12 — Comparison of 2.7kb DNA block of AbSV gemome with corresponding sequence in variant, along with alignment of their encoding amino acid sequences. The AbSV variant was referred by AbSV-m19 and its corresponding ORFs were indicated by mORF16 and mORF17. A, The both shared DNA sequence identity of 63.21%. The Variance of DNA sequence did not result in ORFs termination. The ORF17 protein (B) and C-terminal region of ORF16 protein (C) only shared the 57.78% and 67.31% identities with counterparts of variant sequence respectively. But the C-terminal residues, which were denoted by red line, were conserved. The positions of 2.7kb DNA in genome were signed by numbers. The identical bases/residues were highlighted by asterisks. (0.05 MB PDF) [file pone.0013850.s016.pdf]

A

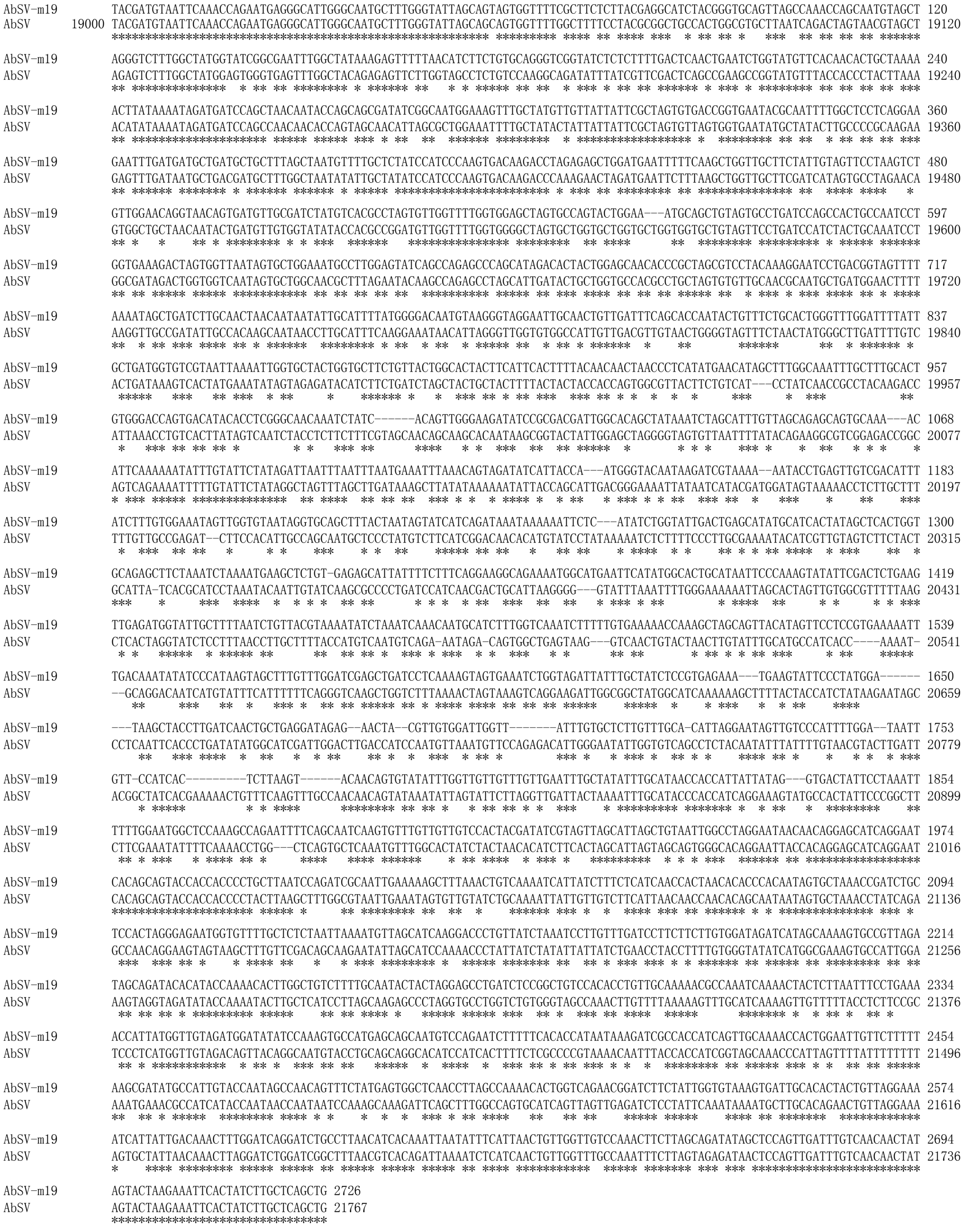

B

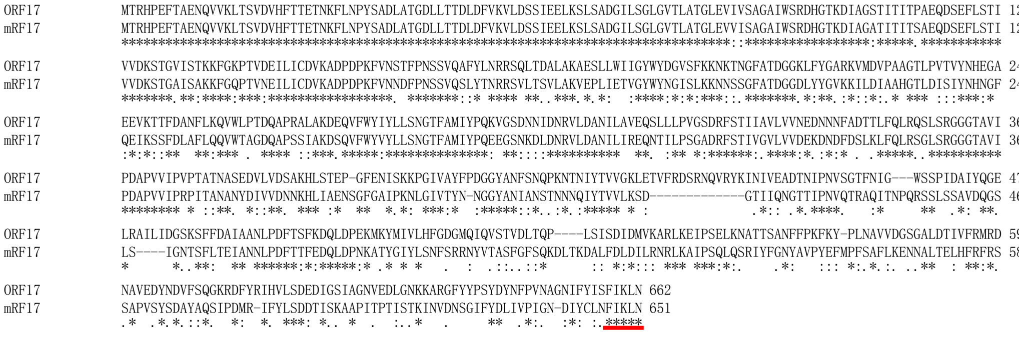

C

```
mRF16        MRALGNALGISSSGFRFSYEASTGAVSQTSNVARVFGYGIGEFGYKEFLTSSVQGRYLSFDSTESGMFTTLLKTYKIDDPANNTSSDIGNGKFAMLLLFASVTGEYAILAPQEEFDDADA 320
ORF16    204 MRALGNALGISSSGFGFSYAAATGVLNQTSNVARVFGYGVGEFGYREFLVASVQGRYLSFDSAEAGMFTTLLKTYKIDDPANNTSSNISAGNFAILLLFASVSGEYAILAPQEEFDNADD 323
             *************** *** *:**.:.*************:*****:***.:***********:*:*********************:*.  *:**:*******:*************:**

mRF16        ALANVLLYPSQVTRPRELDEFFKLVASIVVPKSVGTGNSDVAIYVTPSVGFGGASASTGN-AAVVPDPATANPGERLVVNSAGNALEYQPEPSIDTTGATPASVLQRNPDGSFKIADLAT 439
ORF16        ALANILLYPSQVTRPKELDEFFKLVASIIVPRTVAANNTDVVVYTTPDVGFGGASAGAGAGGAVVPDPSTANPGDRLVVNSAGNALEYKPEPSIDTAGATPASVLQRNADGTFKVADIAT 443
             ****:**********:************:**::*.:.*:**.:*.**.********.:*  .******:*****:*************:*******:***********.**:**:**:**

mRF16        NNNIAFYGDNVRVGIATVDFSTNTVSALGLDFIADGVVIKIGATGASVTGTTSFTFTTTNPHMNIALANLLCTVGPVTYTSGNKS--ITVGKISATIGTAINLAFVSRAVQN-IQKIFVFYRLI 560
ORF16        SNNLAFQGNNIRVGVAIVDVVTGVVSNYGLDFVTDKVTMKYSRDTSSDLATATFTTTTSG-VTSVILSTAYKTIKPVTYSQSTSSFVATASTISGTIGARGSVNFIQKASETGSQKIFVFYRLV 566
             .**:** *:*:***:* **. *..**  ****::* *.:* .    :*  .*::** **:.    .: *:.    *: ****:....*   *...**.***:   .: *:.:* :.   *********:
```
